# Scaffold Simplification Yields Potent Antibacterial Agents That Target Bacterial Topoisomerases

**DOI:** 10.3390/molecules31020240

**Published:** 2026-01-10

**Authors:** Lyubov Khudiakova, Kristina Komarova, Maxim Zhuravlev, Dmitry Deniskin, Alexey Golovanov, Artemiy Nichugovskiy, Kirill Babkin, Maria Zakharova, Mikhail Chudinov, Elizaveta Rogacheva, Lyudmila Kraeva, Olga Shevtsova, Daria Ipatova, Dmitry Skvortsov, Dmitrii Lukianov, Maxim Kryakvin, Maxim Gureev, Alexey Lukin

**Affiliations:** 1 Lomonosov Institute of Fine Chemical Technologies, MIREA—Russian Technological University, Moscow 119454, Russia; 2 Pasteur Institute of Epidemiology and Microbiology, Saint Petersburg 197101, Russia; 3 Department of Chemistry, Lomonosov Moscow State University, GSP-1, 1-3 Leninskiye Gory, Moscow 119991, Russia; 4 Center for Molecular and Cellular Biology, Moscow 121205, Russia; 5 Laboratory of Bio- and Cheminformatics, School of Computer Science, Physics and Technology, HSE University, Saint Petersburg 190121, Russia; 6 Department of Medical Chemistry, Institute of Chemistry, Saint Petersburg State University, Saint Petersburg 199034, Russia

**Keywords:** antibacterial agents, scaffold simplification, amino biphenyls, amino diphenyl ethers, ESKAPE pathogens, DNA replication inhibitors, topoisomerase I, DNA gyrase

## Abstract

This work describes the lead optimization of a promising class of antibacterial compounds, derived from a previously reported N-[4-(4-fluorophenoxy)phenyl]-6-(methylsulfonyl)-2,6-diazaspiro [3.4]octane-8-carboxamide (**LK1819**), through systematic scaffold simplification. A novel series of amide derivatives were designed and synthesized, exploring key structural variations, including the replacement of the diphenyl ether core with a biphenyl system. All compounds were evaluated for in vitro antibacterial activity against the ESKAPE panel of pathogens. The most potent simplified analogs demonstrated exceptional, broad-spectrum activity, with minimum inhibitory concentrations (MICs) that were 10 to 100 times lower than the control antibiotic ciprofloxacin against many strains. Mechanistic studies using a reporter system and enzymatic assays revealed that the compounds do not inhibit protein synthesis but disrupt DNA replication, exhibiting a dose-dependent inhibitory effect on bacterial topoisomerase I and DNA gyrase. The compounds showed moderate toxicity against human cell lines, consistent with their DNA-targeting mechanism, but cytotoxicity assays indicated a sufficient selectivity window. We conclude that scaffold simplification successfully yielded highly potent antibacterial agents with a defined mechanism of action, presenting a promising foundation for further development as antibiotics and potentially as anticancer agents.

## 1. Introduction

**Figure 1:**
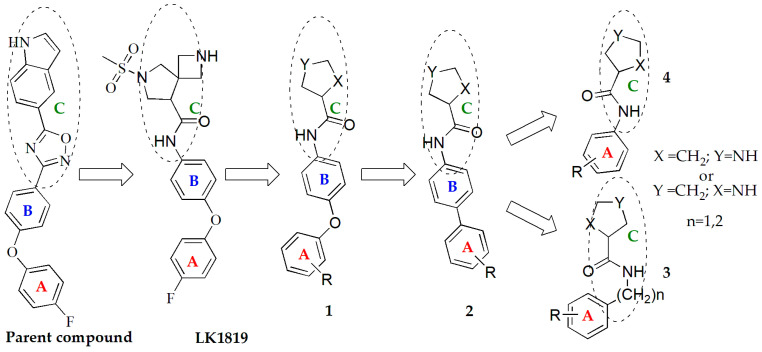
Parent compounds structures of and design of target substances. The scaffold moietys subjected to modification are marked with colored letters. Red (A) is the A ring of the bisaryl fragment, blue (B) is the B ring of the bisaryl fragment, and green (C) is the carboxamide moiety.

The problem of microbial pathogen’s resistance does not seem to have a common solution [1,2,3,4]. Therefore, the search for new antibacterial agents is carried out among the most diverse compound classes [5,6]. New bacteria killers can be found among heteroaromatic [7,8] and spirocyclic compounds [9], peptides [10], and amino acid derivatives [11], and many other substance types [12,13,14]. Both classical methods of medicinal chemistry and generative AI [15,16,17] are used in the search. “Oxadiazole antibiotics”, as named by the authors [18,19], were proposed in 2014. These substances exhibit synergistic activity with oxacillin against multiresistant strains of *S. aureus* (MRSA) [20,21,22] and can be used against other Gram-positive pathogens like *Clostridioides difficile* [23]. Starting with best structure [18], we recently proposed a new design of antibacterial compounds based on the isosteric replacement in the original core [24] (Figure 1). The best substance (**LK1819**) activity among the new series was 10 times higher than the parent structure activity and was also equal to or higher than the activity of the control compound (ciprofloxacin) against pathogens of the ESKAPE panel. Against a panel of MRSA clinical isolates, this substance also showed excellent results. MICs were in the range of 0.33–2.6 μg/mL and, in most cases, were lower than the control MICs. Lead optimization strategy often involves simplifying the scaffold. The series of compounds obtained through further simplification of the **LK1819** structure showed even more impressive results, presented in this work.

## 2. Results

### 2.1. Design of Target Substances

Compound **LK1819** was obtained by isosteric replacement of the oxadiazole ring with an amide group in the parent compound’s structure. Maintaining the planar indole peripheral moiety in the fragment **C** resulted in activity loss, but replacing it with alicyclic amine yielded even more active compounds [24]. The most active analogs of **LK1819** also had a pyrrolidine ring in the **C** fragment. Previous studies of the SAR parameters have shown that the substituents in ring **A** can vary, but should preferably remain hydrophobic [18,25]. These considerations formed the basis for the **1a**–**g** series design.

Replacing the diphenyl ether group (**A**-O-**B**) with biphenyl (**A**-**B**) is the next step in simplification of the scaffold. This modification produced the **2a**–**i** series. The next step was the removal of ring **B** or its replacement by an aliphatic fragment. This led to series **3** and **4**, which were obtained but will not be discussed further in detail due to the activity loss.

### 2.2. Chemistry

**Scheme 1:**
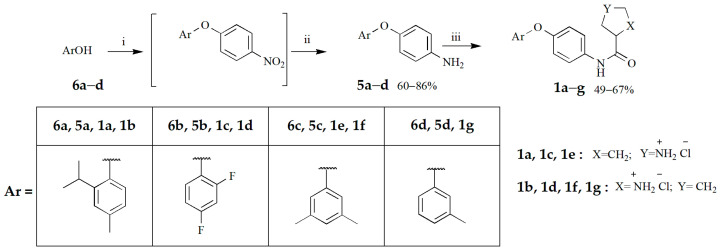
Synthesis of series compounds **1a**–**g**. Reagents and conditions: (i) 1-fluoro-4-nitrobenzene, K_2_CO_3_/DMF, 130 °C, 18 h; (ii) ammonium formate, 10%Pd/C, EtOH, reflux, 2 h; (iii) N-Boc-proline (or N-Boc-isoproline), HBTU, triethylamine, DMF, rt, 12 h.

The substituted 4-phenoxyanilines (**5a**–**d**) required in the synthesis of the **1a**–**g** series were synthesized from phenols **6a**–**d**, followed by acylation with Boc-protected pyrrolidine carboxylic acids to **1a**–**g**, according to Scheme 1.

**Scheme 2:**
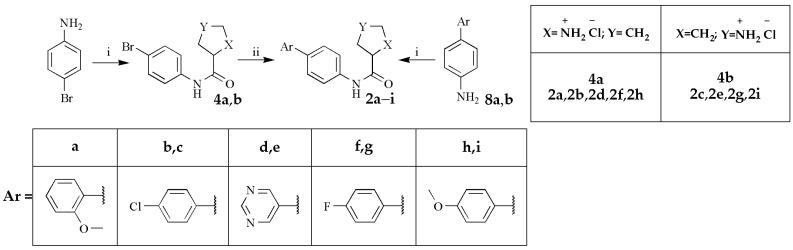
Synthesis of series compounds **2a**–**i**. Reagents and conditions: (i) N-Boc-proline (or N-Boc-isoproline), HBTU, triethylamine, DMF, rt, 12 h; (ii) corresponding arylboronic acid, Cs_2_CO_3_, [bis (diphenylphosphino)ferrocene] dichloro palladium (II), dioxane–water (10:1), 105 °C, 6 h.

Substances of series **2a**–**i** were obtained according to Scheme 2 by two different ways.

The first route involved acylation of commercially available substituted amino biphenyls **8a**,**b** with Boc-protected proline (or isoproline) followed by removal of the protecting group to synthesize compounds **2a**–**c**. In the second route, p-bromoaniline was acylated with Boc-protected pyrrolidine carboxylic acids to form *tert*-butyl 2-{[(4-bromophenyl)amino]carbonyl}pyrrolidine-1-carboxylate (**4a**) and *tert*-butyl 3-{[(4-bromophenyl)amino]carbonyl}pyrrolidine-1-carboxylate (**4b**); then, anilides **4** were introduced into a Suzuki reaction with substituted boronic acids, followed by deprotection to yield compounds **2d**–**i**.

### 2.3. Antibacterial Activity Evaluation

All synthesized compounds were tested against Gram-positive (*S. aureus* and *E. faecium*) or Gram-negative (*P. aeruginosa*, *A. baumannii*, *K. pneumoniae*, *E. cloacae*) pathogens of the so-called ESKAPE panel [26]. Clinically used antibiotic ciprofloxacin was used as positive control and comparator. The compounds were initially screened at a single concentration to determine the presence and the diameter of the bacterial growth inhibition zone around the drug-treated disk (Table S1). Those compounds that displayed growth inhibition were tested in serial dilution mode to determine the minimum inhibitory concentration (MIC) (Table 1).

As follows from the presented data, almost all tested compounds exhibited in vitro broad-spectrum antibacterial activity equal to, and in some cases significantly exceeding, the ciprofloxacin activity.

### 2.4. Discovery of the Antibacterial Action Mechanism

**Figure 2:**
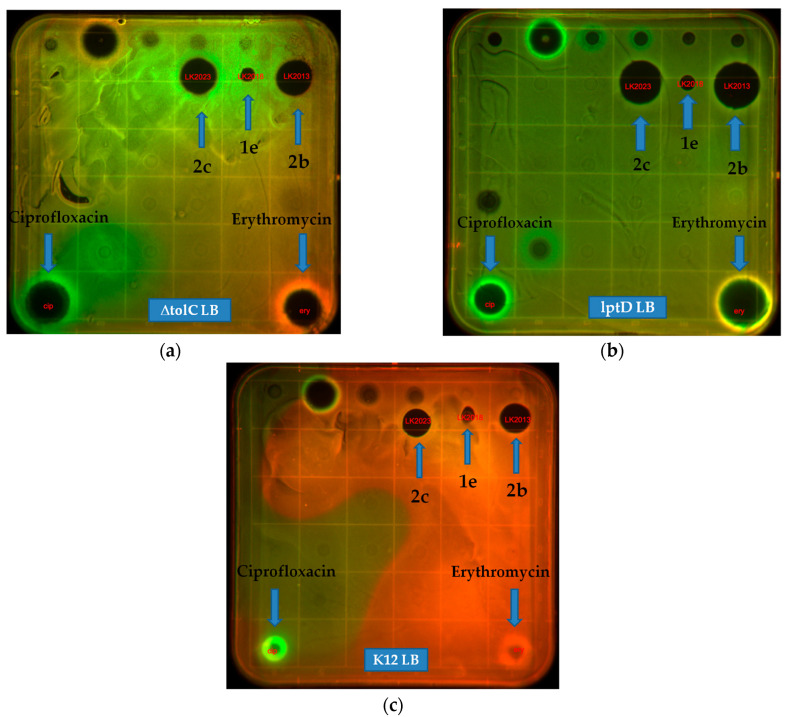
Screening of antibacterial activity mechanism of the test compounds **1e**, **2c**, and **2b** using the pDualrep2 reporter system. Shown is an agar plate containing a lawn of *E. coli* transformed with the pDualrep2 plasmid and 2 µL spots of compounds tested at a concentration of 20 µg/mL. Erythromycin (5 μg/mL) and ciprofloxacin (2.5 μg/mL) 2 μL spots were used as controls. Red fluorescence is induced by translation-stalling compounds, and green fluorescence is induced by substances that damage DNA. (**a**) ΔtolC strain; (**b**) lptD strain; (**c**) K12 strain.

The action mechanism of most active compounds **1e**, **2b**, and **2c** was evaluated using the pDualrep2 reporter system [27] in *E. coli* K12, ΔtolC [28], and lptD^mut^ [29] strains transformed with the corresponding plasmid. All compounds demonstrated antimicrobial activity, confirmed by the formation of growth inhibition zones of the test strains. The obtained data are presented in Figure 2.

**Figure 3:**
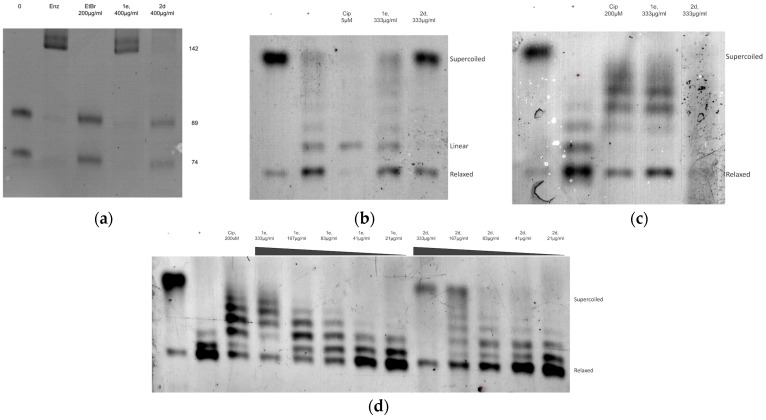
The influence of the studied substances on the enzymes functioning: (**a**) Klenow fragment polymerization; (**b**) DNA gyrase relaxation; (**c**) topoisomerase I relaxation; and (**d**) topoisomerase I relaxation with different concentrations of **2c**.

Given that tested compounds elicited an SOS response in bacteria, it was decided to test their effect on bacterial topoisomerases and DNA polymerase as potential targets. Both series’ most active substances (**1e** and **2c**) were tested against *E. coli* topoisomerase I (TopA), DNA gyrase, topoisomerase IV, and Klenow fragment. The obtained data are presented in Figure 3.

### 2.5. Toxicity Study

**Figure 4:**
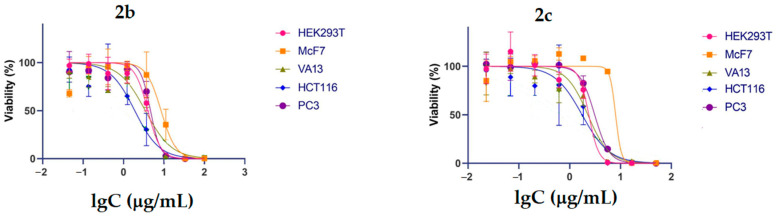
MTT test results of the compounds **2b** and **2c** against six human cell lines.

The cytotoxicity of active compounds **2b** and **2c** was evaluated by the MTT test. The action of these two compounds for cell lines appears similar. MCF7 breast cancer cells showed the least sensitivity to both tested compounds, with an IC50abs value of approximately 8 μg/mL for both substances. Other cell lines were the most sensitive, with an IC50abs value in the range 1.8–2.1 μg/mL for compound **2b** and 1.9–4.7 μg/mL for compound **2c**. The observed effects on the bacterial cells (Table 1) were slightly more pronounced than that of the human cell lines. The obtained data are presented in Figure 4 and Table S2.

### 2.6. In Silico Studies

Initially, we considered as the main target penicillin-binding protein Pbp2a of *S. aureus* [30], identified in this role for the parent compound [31]. Five active compounds (**1b**, **1e**, **2b**, **2c**, and **2f**) were selected for calculations. The binding poses of the ligands were predicted using the induced-fit docking method. The docking results are given in Table 2.

Only compound **1e** shows weak affinity for the Pbp2a. However, significant structural differences between the studied substances and the parent compound suggest a completely different action mechanism. In vitro testing results as well as several studies [32,33,34] suggest that the targets of the synthesized structures may be DNA replication enzymes, like gyrases and topoisomerases. Experimental and AlphaFold-calculated models of target proteins, DNA topoisomerase 1 (TopA) and DNA gyrase (GyrA + GyrB), were found for ESKAPE pathogens. In the TopA case, experimental data exist for *S. aureus* only; hypothetical structures have been found for the remaining microorganisms. More confirmed structures found for DNA gyrase. Unfortunately, protein models constructed using machine learning methods can differ from the real protein structure, especially if experimentally described homologs are absent. Therefore, the predictive power of modeling ligand–protein complexes based on these structures will be strongly limited. For this reason, only models with experimental data were selected for calculations.

## 3. Discussion

As evidenced by the results of antibacterial activity measurements, simplification of the structure of the compound **LK1819** led to compounds 10–100 times more active than ciprofloxacin against ESKAPE pathogens. The limited number of synthesized substances precludes a comprehensive SAR study, especially for compounds of series **1a**–**g**. However, a number of conclusions can be drawn based on a comparison of the activity of the target compounds.

If we include into consideration the previously described [24] **LK1823** (N-[4-(4-fluorophenoxy)phenyl]pyrrolidine-3-carboxamide) (MIC’s 0.33–2.6 μg/mL against ESKAPE pathogens) and **LK1828** (N-[4-(4-fluorophenoxy)phenyl]pyrrolidine- 2-carboxamide) (MIC’s 0.39–6.2 μg/mL), which structurally belong to series **1**, then a trend of increasing antibacterial properties with increasing lipophilicity of fragment A is noticeable.

Replacing the phenyl ring A with a heteroaromatic pyrimidine ring in compounds **2d** and **2e** results in complete loss of activity. The electron-donating methoxy group at position 2 of ring A (**2a**) acts similarly. Methoxy groups at the para-position (**2h** and **2i**) lead to a 10-fold reduction in activity. The best antimicrobial activity in the **2a**–**i** series is associated with para-halogen substituents (**2b**, **2c**, and **2f**). The role of the nitrogen atom position in fragment C remains unclear, as do the reasons for the activity loss of compound **1c**.

In silico data analysis shows that the compounds interact weakly or moderately with all available targets. Compound **1e** exhibits affinity for Pbp2a, and **2c** exhibits affinity for GyrA/B from *S. aureus*. Compound **2b** is capable of binding well to GyrA/B from *A. baumannii* and *P. aeruginosa*; however, the scoring function values for this compound are on the borderline of low activity. Analysis using the pDualrep2 reporter system also shows that the substances do not affect protein biosynthesis in bacterial cells but disrupt nucleic acid replication. At a high concentration of 400 μg/mL, compound **2c** partially inhibits the Klenow fragment, as evidenced by the smaller amount of product with a length of 142 compared to the control line (Figure 3a). Compound **2c** also inhibits DNA gyrase relaxation (the amount of supercoiled DNA is similar to the control without enzyme, Figure 3b) and, like compound **2b**, inhibits topoisomerase I relaxation (there is a lesser amount of relaxed DNA product than in the control with enzyme, Figure 3c). Moreover, this activity is concentration-dependent, as shown in Figure 3d. A lower concentration of the substance leads to a more complete relaxation of DNA, making it more similar to the control line with an enzyme. The studied substances do not have a significant effect on topoisomerase IV.

A noticeable lack of correlation is observed between the high in vitro efficiency and the relatively low docking values (−4.0 to −6.5 kcal/mol) to TopA. The scoring function values in this range typically indicate weak binding. This parameter is likely inappropriate for action mechanisms involving DNA intercalation, as the model does not include the DNA molecule itself.

A closer examination of the MIC against most ESKAPE pathogens and IC_50_ ratio reveals that the therapeutic window for compounds **2b** and **2c** is approximately tenfold. The proposed mechanism of action for intercalators presents a potential safety concern for their development as antibacterial drugs. The substances described have a profile that makes them candidates for anti-tumor drugs, similar to actinomycin D, an intercalator that is effective against bacteria but has a narrow therapeutic window [35]. However, actinomycin D’s therapeutic window is still notably narrower than tenfold, which suggests that compounds **2b** and **2c** could still remain viable as antibiotic candidates. On the other hand, these substances could be considered for inclusion in the “last line of defense” antibiotics against multidrug-resistant bacteria, where the risk of infection outweighs the risk of drug toxicity, much like the neuro- and nephrotoxic antibiotic colistin [36,37]. The problem of a narrow therapeutic window in this case may be partially solved by implementing therapeutic drug monitoring during clinical use. Moreover, the ongoing improvement in these compounds could expand their therapeutic scope via molecular modification, as demonstrated for colistin [38].

## 4. Materials and Methods

### 4.1. General Chemistry

All commercial reagents were used without purification. NMR spectra were recorded using a DPX-300 spectrometer (Bruker, Billerica, MA, USA) (^1^H: 300 MHz; ^13^C: 75 MHz). Chemical shifts are reported as parts per million (δ, ppm). The residual solvent peak (CHCl_3_ or DMSO-*d6*) was used as the internal standard: 7.28 or 2.51 for ^1^H and 77.07 or 40.00 ppm for ^13^C. Multiplicities are abbreviated as follows: s = singlet, d = doublet, t = triplet, q = quartet, m = multiplet, br = broad, dd = doublet of doublets, dt = doublet of triplets, ddd = doublet/doublets of doublets (see Supplementary Materials). Coupling constants, J, are reported in Hz. Mass spectra were recorded using a Bruker microTOF spectrometer (Bruker, Billerica, MA, USA) (ionization by electrospray, positive ion detection). Analytical thin-layer chromatography was carried out on UV-254 silica gel plates (Imid LLC, Krasnodar, Russia) using appropriate eluents. Compounds were visualized with short-wavelength UV light. Column chromatography was performed using silica gel Merck grade 60 (0.040−0.063 mm) 230−400 mesh (Merck & Co., Inc., Rahway, NJ, USA).

### 4.2. Synthesis

#### 4.2.1. General Procedure A for Phenoxyphenyl Amines **5a**–**d**

To a solution of 4-fluoronitrobenzene (1.00 g, 7.09 mmol) and corresponding phenol **6** (1.1 eqv, 7.79 mmol) in DMF (25 mL) K_2_CO_3_ was added (1.96 g, 14.2 mmol, 2 eqv), and the resulting mixture was stirred at 120 °C for 12 h. The reaction mixture was cooled to room temperature, poured into water (50 mL), and extracted with ethyl acetate (3 × 30 mL). The organic extracts were combined, the solvent was removed in vacuo, and the residue was dissolved in EtOH (40 mL). Then, to this solution, ammonium formate (1.68 g, 35.7 mmol, 5 eqv) and 10% Pd/C (5% mass, 0.091 g) was added, and the resulting mixture was heated under reflux for 2 h. Then, the reaction mixture was filtrated through Celite layer, the filtrate was evaporated, and the residue was dissolved in ethyl acetate (40 mL). The solution was extracted two times with water (2 × 20 mL). Organic phase was dried over anhydrous Na_2_SO_4_, and the solvent was removed in vacuo to give amine **5**.

4-(2-isopropyl-4-methylphenoxy)aniline (**5a**). The compound was synthesized according to General procedure A from 2-isopropyl-4-methylphenol **6a** (1.31 g). Yield 1.47 g (76%), gray solid. ^1^H NMR (300 MHz, DMSO-*d6*) δ 7.16 (d, *J* = 7.8 Hz, 1H), 6.81 (d, *J* = 6.8 Hz, 1H), 6.71–6.64 (m, 2H), 6.60–6.54 (m, 2H), 6.47–6.43 (m, 1H), 4.91 (s, 2H), 3.23 (hept, *J* = 6.8 Hz, 1H), 2.15 (s, 3H), 1.18 (d, *J* = 6.9 Hz, 6H). ^13^C NMR (75 MHz, DMSO-*d6*) δ 155.31, 146.98, 144.78, 136.04, 134.74, 126.30, 123.16, 119.86, 117.27, 114.97, 26.29, 22.87, 20.69. HRMS (ESI) *m*/*z* calcd for C_16_H_20_NO (M + H^+^) 242.1545, found 242.1545.

4-(2,4-difluorophenoxy)aniline (**5b**). The compound was synthesized according to General procedure A from 2,4-difluorophenol **6b** (1.26 g). Yield 1.20 g (62%), gray solid. ^1^H NMR (300 MHz, DMSO-*d6*) δ 7.41–7.32 (m, 1H), 7.03–6.94 (m, 2H), 6.77–6.71 (m, 2H), 6.60–6.55 (m, 2H), 4.95 (s, 2H). ^13^C NMR (75 MHz, DMSO-*d6*) δ 157.14 (dd, *J* = 241.4, 10.7 Hz), 152.80 (dd, *J* = 248.3, 12.6 Hz), 146.71, 145.48, 142.17 (d, *J* = 10.1 Hz), 120.60 (d, *J* = 9.6 Hz), 119.33, 115.13, 111.70 (d, *J* = 24.7 Hz), 105.53 (dd, *J* = 27.3, 22.3 Hz). HRMS (ESI) *m*/*z* calcd for C_12_H_10_F_2_NO (M + H^+^) 222.0730, found 222.0731.

4-(3,5-dimethylphenoxy)aniline (**5c**). The compound was synthesized according to General procedure A from 3,5-dimethylphenol **6b** (0.95 g). Yield 0.90 g (60%), light brown solid. ^1^H NMR (300 MHz, DMSO-*d6*) δ 6.76–6.70 (m, 2H), 6.64–6.61 (m, 1H), 6.61–6.55 (m, 2H), 6.47–6.43 (m, 2H), 4.97 (s, 2H), 2.19 (s, 6H). ^13^C NMR (75 MHz, DMSO-*d6*) δ 176.90, 167.06, 159.75, 153.31, 137.81, 129.24, 119.96, 118.30, 20.40. HRMS (ESI) *m*/*z* calcd for C_14_H_16_NO (M + H^+^) 214.1232, found 214.1235.

4-(*m*-tolyloxy)aniline (**5d**). The compound was synthesized according to General procedure A from m-cresol **6d** (1.26 g). Yield 1.27 g (60%), light brown solid. ^1^H NMR (300 MHz, DMSO-*d6*) δ 7.16 (t, *J* = 7.7 Hz, 1H), 6.84–6.72 (m, 3H), 6.68–6.55 (m, 4H), 4.98 (s, 2H), 2.24 (s, 3H). 7.16 (t, *J* = 7.7 Hz, 1H), 6.83–6.78 (m, 1H), 6.78–6.71 (m, 2H), 6.68–6.55 (m, 4H), 4.98 (s, 2H), 2.24 (s, 3H). ^13^C NMR (75 MHz, DMSO-*d6*) δ 159.41, 145.87, 145.81, 139.60, 129.78, 122.83, 121.36, 117.26, 115.25, 113.89, 21.41. HRMS (ESI) *m*/*z* calcd for C_13_H_14_NO (M + H^+^) 200.1075, found 200.1075.

#### 4.2.2. General Synthesis Procedure B for Compounds **1a**–**g**, **2a**–**c** and **4a**–**b**

To a solution of N-(*tert*-butoxycarbonyl)proline (or N-(*tert*-butoxycarbonyl)isoproline) (0.248 g, 1.15 mmol, 1 eqv) in DMF (10 mL), HBTU (0.523 g, 1.38 mmol, 1.2 eqv), p-bromoaniline (0.18 g, 1.04 mmol, 0.9 eqv) (for **4a**–**b**) or aniline **5** (for **1a**–**g**) or **8** (for **2a**–**c**), and Et_3_N (0.139 g, 1.38 mmol, 1.2 eqv) were added successively. The resulting mixture was stirred at room temperature for 12 h. The reaction mixture was poured into water (25 mL) and extracted with ethyl acetate (2 × 10 mL). Combined organic phases were washed with 1% aqueous citric acid (2 × 5 mL) and with saturated NaHCO_3_ solution (2 × 5 mL). The organic phase was dried over anhydrous Na_2_SO_4_, and the solvent was removed *in vacuo*. The residue was purified by column chromatography on silica gel eluting with 0% to 2% methanol in CH_2_Cl_2_. Combined fractions were evaporated in vacuo. The *tert*-butyloxycarbonyl protecting group was removed using 4M HCl in dioxane (5 mL). The resulting precipitate was recrystallized from EtOH.

N-(4-bromophenyl)pyrrolidine-2-carboxamide hydrochloride (**4a**) and N-(4-bromophenyl)pyrrolidine-2-carboxamide hydrochloride (**4b**) were synthesized according to General procedure B. The NMR data of the compounds correspond to those described previously [39,40].

N-(4-(2-isopropyl-4-methylphenoxy)phenyl)pyrrolidine-3-carboxamide hydrochloride (**1a**). The compound was synthesized according to General procedure B from aniline **5a** (0.25 g). Yield 0.21 g (49%), white solid. ^1^H NMR (300 MHz, DMSO-*d6*) δ 10.43 (s, 1H), 9.50 (s, 1H), 9.22 (s, 1H), 7.65–7.57 (m, 2H), 7.24 (d, *J* = 7.8 Hz, 1H), 6.94 (d, *J* = 8.0 Hz, 1H), 6.91–6.84 (m, 2H), 6.64–6.59 (m, 1H), 3.36–3.08 (m, 6H), 2.31–2.22 (m, 1H), 2.20 (s, 3H), 2.12–1.94 (m, 1H), 1.15 (d, *J* = 6.9 Hz, 6H). ^13^C NMR (75 MHz, DMSO-*d6*) δ 170.33, 153.86, 153.65, 136.93, 136.43, 134.29, 127.16, 125.21, 121.47, 119.92, 118.10, 47.19, 45.16, 43.34, 29.30, 26.72, 23.27, 20.89. HRMS (ESI) *m*/*z* calcd for C_21_H_27_N_2_O_2_ (M + H^+^) 339.2073, found 339.2072.

N-(4-(2-isopropyl-4-methylphenoxy)phenyl)pyrrolidine-2-carboxamide hydrochloride (**1b**). The compound was synthesized according to General procedure B from aniline **5a** (0.25 g). Yield 0.19 g (44%), white solid. ^1^H NMR (300 MHz, DMSO-*d6*) δ 10.88 (s, 1H), 10.00 (s, 1H), 8.67 (s, 1H), 7.65–7.58 (m, 2H), 7.25 (d, *J* = 7.9 Hz, 1H), 6.98–6.94 (m, 1H), 6.93–6.88 (m, 2H), 6.66–6.62 (m, 1H), 4.45–4.32 (m, 1H), 3.31–3.21 (m, 2H), 3.18–3.07 (m, 1H), 2.46–2.36 (m, 1H), 2.21 (s, 3H), 2.01–1.89 (m, 3H), 1.15 (d, *J* = 6.9 Hz, 6H). ^13^C NMR (75 MHz, DMSO-*d6*) δ 166.88, 154.35, 153.42, 137.02, 136.52, 133.47, 127.22, 125.40, 121.61, 120.11, 118.15, 59.88, 46.05, 30.12, 26.69, 24.00, 23.30, 20.89. HRMS (ESI) *m*/*z* calcd for C_21_H_27_N_2_O_2_ (M + H^+^) 339.2073, found 339.2073.

N-(4-(2,4-difluorophenoxy)phenyl)pyrrolidine-3-carboxamide hydrochloride (**1c**). The compound was synthesized according to General procedure B from aniline **5b** (0.25 g). Yield 0.222 g (50%), white solid. ^1^H NMR (300 MHz, DMSO-*d6*) δ 10.47 (s, 1H), 9.48 (s, 1H), 9.20 (s, 1H), 7.66–7.59 (m, 2H), 7.52–7.42 (m, 1H), 7.27–7.17 (m, 1H), 7.15–7.07 (m, 1H), 6.98–6.92 (m, 2H), 3.34–3.16 (m, 5H), 2.30–2.18 (m, 1H), 2.08–1.97 (m, 1H). ^13^C NMR (75 MHz, DMSO-*d6*) δ 170.40, 158.39 (dd, *J* = 242.8, 10.8 Hz), 153.79 (dd, *J* = 249.3, 13.1 Hz), 153.05, 140.14 (d, *J* = 11.8 Hz), 134.94, 123.12 (d, *J* = 9.9 Hz), 121.38, 117.44, 112.41 (dd, *J* = 22.9, 3.8 Hz), 106.07 (dd, *J* = 27.5, 22.2 Hz), 47.05, 45.11, 43.33, 29.34. HRMS (ESI) *m*/*z* calcd for C_17_H_17_F_2_N_2_O_2_ (M + H^+^) 319.1258, found 319.1258.

N-(4-(2,4-difluorophenoxy)phenyl)pyrrolidine-2-carboxamide hydrochloride (**1d**). The compound was synthesized according to General procedure B from aniline **5b** (0.25 g). Yield 0.268 g (60%), white solid. ^1^H NMR (300 MHz, DMSO-*d6*) δ 10.99 (s, 1H), 10.08 (s, 1H), 8.67 (s, 1H), 7.68–7.60 (m, 2H), 7.55–7.42 (m, 1H), 7.29–7.20 (m, 1H), 7.16–7.08 (m, 1H), 7.02–6.95 (m, 2H), 4.38 (s, 1H), 3.26 (s, 2H), 2.48–2.35 (m, 1H), 2.00–1.88 (m, 3H). ^13^C NMR (75 MHz, DMSO-*d6*) δ 166.96, 158.50 (dd, *J* = 243.1, 10.8 Hz), 153.53, 153.34 (dd, *J* = 246.5, 13.4 Hz), 139.97 (d, *J* = 10.7 Hz), 134.20, 123.31 (d, *J* = 10.2 Hz), 121.57, 117.50, 112.47 (d, *J* = 23.2 Hz), 106.13 (dd, *J* = 27.3, 22.6 Hz), 59.83, 46.00, 30.16, 24.00. HRMS (ESI) *m*/*z* calcd for C_17_H_17_F_2_N_2_O_2_ (M + H^+^) 319.1258, found 319.1258.

N-(4-(3,5-dimethylphenoxy)phenyl)pyrrolidine-3-carboxamide hydrochloride (**1e**). The compound was synthesized according to General procedure B from aniline **5c** (0.25 g). Yield 0.218 g (48%), white solid. ^1^H NMR (300 MHz, DMSO-*d6*) δ 10.46 (s, 1H), 9.44 (s, 1H), 9.18 (s, 1H), 7.67–7.60 (m, 2H), 7.00–6.95 (m, 2H), 6.73 (s, 1H), 6.56 (s, 2H), 3.35–3.15 (m, 5H), 2.32–2.25 (m, 1H), 2.22 (s, 6H), 2.10–2.01 (m, 1H). ^13^C NMR (75 MHz, DMSO-*d6*) δ 170.39, 157.75, 152.46, 139.63, 135.06, 124.93, 121.41, 119.81, 115.88, 47.12, 45.14, 43.37, 29.32, 21.25. HRMS (ESI) *m*/*z* calcd for C_19_H_23_N_2_O_2_ (M + H^+^) 311.1760, found 311.1760.

N-(4-(3,5-dimethylphenoxy)phenyl)pyrrolidine-2-carboxamide hydrochloride (**1f**). The compound was synthesized according to General procedure B from aniline **5c** (0.25 g). Yield 0.247 g (55%), white solid. ^1^H NMR (300 MHz, DMSO-*d6*) δ 10.94 (s, 1H), 9.99 (s, 1H), 8.68 (s, 1H), 7.70–7.60 (m, 2H), 7.06–6.96 (m, 2H), 6.75 (s, 1H), 6.57 (s, 2H), 4.38 (s, 1H), 3.27 (s, 2H), 2.45–2.37 (m, 1H), 2.23 (s, 6H), 2.01–1.89 (m, 3H). ^13^C NMR (75 MHz, DMSO-*d6*) δ 166.96, 157.55, 152.96, 139.70, 134.23, 125.09, 121.55, 119.93, 116.03, 59.92, 46.06, 30.12, 24.00, 21.28. HRMS (ESI) *m*/*z* calcd for C_19_H_23_N_2_O_2_ (M + H^+^) 311.1760, found 311.1761.

N-(4-(m-tolyloxy)phenyl)pyrrolidine-2-carboxamide hydrochloride (**1g**). The compound was synthesized according to General procedure B from aniline **5d** (0.25 g). Yield 0.20 g (44%), white solid. ^1^H NMR (300 MHz, DMSO-*d6*) δ 10.98 (s, 1H), 10.05 (s, 1H), 8.68 (s, 1H), 7.70–7.62 (m, 2H), 7.25 (t, *J* = 7.7 Hz, 1H), 7.05–6.98 (m, 2H), 6.96–6.90 (m, 1H), 6.82–6.70 (m, 2H), 4.45–4.34 (m, 1H), 3.33–3.21 (m, 2H), 2.47–2.35 (m, 1H), 2.28 (s, 3H), 2.01–1.89 (m, 3H). ^13^C NMR (75 MHz, DMSO-*d6*) δ 166.67, 157.23, 152.60, 139.80, 134.00, 129.83, 123.98, 121.28, 119.61, 118.59, 115.18, 59.63, 45.77, 29.82, 23.70, 21.05. HRMS (ESI) *m*/*z* calcd for C_18_H_21_N_2_O_2_ (M + H^+^) 297.1603, found 297.1603.

N-(2′-methoxy-[1,1′-biphenyl]-4-yl)pyrrolidine-2-carboxamide hydrochloride (**2a**). The compound was synthesized according to General procedure B from 2′-methoxybiphenyl-4-amine **8a** (0.25 g). Yield 0.12 g (26%), white solid. ^1^H NMR (300 MHz, DMSO-*d6*) δ 11.13 (s, 1H), 10.13 (s, 1H), 8.72 (s, 1H), 7.78–7.66 (m, 4H), 7.39–7.32 (m, 1H), 7.24–7.16 (m, 2H), 6.95–6.88 (m, 1H), 4.45 (s, 1H), 3.81 (s, 3H), 3.32–3.24 (m, 2H), 2.49–2.38 (m, 1H), 2.02–1.91 (m, 3H). ^13^C NMR (75 MHz, DMSO-*d6*) δ 167.24, 160.17, 141.40, 138.18, 135.99, 130.35, 127.54, 120.22, 119.06, 113.19, 112.24, 60.00, 55.53, 46.07, 30.16, 24.01. HRMS (ESI) *m*/*z* calcd for C_18_H_21_N_2_O_2_ (M + H^+^) 297.1603, found 297.1604.

N-(4′-chloro-[1,1′-biphenyl]-4-yl)pyrrolidine-2-carboxamide hydrochloride (**2b**). The compound was synthesized according to General procedure B from 4′-chlorobiphenyl-4-amine **8b** (0.25 g). Yield 0.18 g (39%), white solid. ^1^H NMR (300 MHz, DMSO-*d6*) δ 11.20 (s, 1H), 10.15 (s, 1H), 8.72 (s, 1H), 7.81–7.74 (m, 2H), 7.72–7.64 (m, 4H), 7.52–7.46 (m, 2H), 4.46 (s, 1H), 3.28 (s, 2H), 2.49–2.38 (m, 1H), 2.00–1.90 (m, 3H). ^13^C NMR (75 MHz, DMSO-*d6*) δ 167.29, 138.71, 138.41, 134.70, 132.41, 129.24, 128.43, 127.43, 120.31, 60.00, 46.07, 30.16, 24.00. HRMS (ESI) *m*/*z* calcd for C_17_H_18_ClN_2_O (M + H^+^) 301.1108, found 301.1108.

N-(4′-chloro-[1,1′-biphenyl]-4-yl)pyrrolidine-2-carboxamide hydrochloride (**2c**). The compound was synthesized according to General procedure B from 4′-chlorobiphenyl-4-amine **8b** (0.25 g). Yield 0.31 g (67%), white solid. ^1^H NMR (300 MHz, DMSO-*d6*) δ 10.58 (s, 1H), 9.47 (s, 1H), 9.20 (s, 1H), 7.78–7.71 (m, 2H), 7.70–7.62 (m, 4H), 7.51–7.46 (m, 2H), 3.38–3.30 (m, 3H), 3.26–3.16 (m, 2H), 2.33–2.21 (m, 1H), 2.11–2.01 (m, 1H). ^13^C NMR (75 MHz, DMSO-*d6*) δ 170.47, 138.73, 138.48, 133.86, 131.97, 128.92, 128.06, 126.97, 119.86, 46.85, 44.91, 43.17, 29.02. HRMS (ESI) *m*/*z* calcd for C_17_H_18_ClN_2_O (M + H^+^) 301.1108, found 301.1108.

#### 4.2.3. General Synthesis Procedure C for Compounds **2e**–**i**

The corresponding arylboronic acid (0.68 mmol, 1.25 eqv) and compound **4a** (0.2 g, 0.54 mmol) (for **2d**,**f**,**h** synthesis) or **4b** (for **2e**,**g**,**i**), [(C_5_H_4_P(C_6_H_5_)_2_)_2_Fe]PdCl_2_·CH_2_Cl_2_ (0.04 g, 0.054 mmol), and Cs_2_CO_3_ (0.352 g, 1.08 mmol) were dissolved in a 100 mL screw cap vial under argon atmosphere in degassed dioxane/water 10:1 mixture (10 mL). The mixture was heated for 6 h at 105 ◦C, and then it was poured in water (50 mL) and extracted with ethyl acetate (2 × 50 mL). Combined organic phases were dried over anhydrous Na_2_SO_4_, and the solvent was removed in vacuo. The residue was purified by column chromatography on silica gel eluting with 0% to 2% methanol in CH_2_Cl_2_. The *tert*-butyloxycarbonyl protecting group was removed using 4M HCl in dioxane (5 mL). The resulting precipitate was recrystallized from EtOH.

N-(4-(pyrimidin-5-yl)phenyl)pyrrolidine-2-carboxamide hydrochloride (**2d**). The compound was synthesized according to General procedure C from pyrimidin-5-ylboronic acid (0.085 g). Yield 0.08 g (48%), white solid. ^1^H NMR (300 MHz, DMSO-*d6*) δ 11.41 (s, 1H), 10.28 (s, 1H), 9.17 (s, 3H), 8.74 (s, 1H), 7.84 (s, 4H), 4.52–4.43 (m, 1H), 3.28 (s, 2H), 2.49–2.40 (m, 1H), 2.01–1.90 (m, 3H). ^13^C NMR (75 MHz, DMSO-*d6*) δ 167.12, 156.50, 154.30, 139.17, 132.75, 128.92, 127.48, 120.11, 59.67, 45.72, 29.82, 23.65. HRMS (ESI) *m*/*z* calcd for C_15_H_16_N_4_O (M + H^+^) 269.1404, found 269.1402.

N-(4-(pyrimidin-5-yl)phenyl)pyrrolidine-3-carboxamide hydrochloride (**2e**). The compound was synthesized according to General procedure C from pyrimidin-5-ylboronic acid (0.085 g). Yield 0.10 g (61%), white solid. ^1^H NMR (300 MHz, DMSO-*d6*) δ 10.82 (s, 1H), 9.65 (s, 1H), 9.39 (s, 1H), 9.16 (s, 3H), 7.88–7.73 (m, 4H), 3.71–3.63 (m, 1H), 3.50–3.31 (m, 4H), 2.37–2.19 (m, 1H), 2.15–1.94 (m, 1H). ^13^C NMR (75 MHz, DMSO-*d6*) δ 170.47, 156.42, 154.14, 139.76, 132.76, 128.26, 127.23, 119.83, 46.59, 44.69, 43.11, 28.88. HRMS (ESI) *m*/*z* calcd for C_15_H_16_N_4_O (M + H^+^) 269.1404, found 269.1406.

N-(4′-fluoro-[1,1′-biphenyl]-4-yl)pyrrolidine-2-carboxamide hydrochloride (**2f**). The compound was synthesized according to General procedure C from 4-fluorophenylboronic acid (0.095 g). Yield 0.10 g (58%), white solid. ^1^H NMR (300 MHz, DMSO-*d6*) δ 11.20 (s, 1H), 10.21 (s, 1H), 8.72 (s, 1H), 7.77 (d, *J* = 8.4 Hz, 1H), 7.71–7.62 (m, 6H), 7.27 (t, *J* = 8.8 Hz, 2H), 4.45 (s, 1H), 3.28 (s, 2H), 2.50–2.38 (m, 1H), 2.01–1.89 (m, 3H). ^13^C NMR (75 MHz, DMSO-*d6*) δ 166.93, 161.75 (d, *J* = 244.3 Hz), 137.74, 136.04, 134.70, 128.33 (d, *J* = 8.2 Hz), 127.08, 119.89, 115.76 (d, *J* = 21.3 Hz), 59.63, 45.72, 29.87, 23.69. HRMS (ESI) *m*/*z* calcd for C_17_H_18_FN_2_O (M + H^+^) 285.1403, found 285.1403.

N-(4′-fluoro-[1,1′-biphenyl]-4-yl)pyrrolidine-3-carboxamide hydrochloride (**2g**). The compound was synthesized according to General procedure C from 4-fluorophenylboronic acid (0.095 g). Yield 0.11 g (63%), white solid. ^1^H NMR (300 MHz, DMSO-*d6*) δ 10.64 (s, 1H), 9.62 (s, 1H), 9.34 (s, 1H), 7.79–7.51 (m, 6H), 7.25 (t, *J* = 8.3 Hz, 2H), 3.40–3.09 (m, 5H), 2.40–2.18 (m, 1H), 2.15–1.94 (m, 1H). ^13^C NMR (75 MHz, DMSO-*d6*) δ 170.27, 161.58 (d, *J* = 243.8 Hz), 138.37, 136.07, 134.04, 128.17 (d, *J* = 7.5 Hz), 126.83, 119.66, 115.66 (d, *J* = 21.2 Hz), 46.65, 44.75, 43.09, 29.00. HRMS (ESI) *m*/*z* calcd for C_17_H_18_FN_2_O (M + H^+^) 285.1403, found 285.1402.

N-(4′-methoxy-[1,1′-biphenyl]-4-yl)pyrrolidine-2-carboxamide hydrochloride (**2h**). The compound was synthesized according to General procedure C from 4-methoxyphenylboronic acid (0.14 g). Yield 0.115 g (47%), white solid. ^1^H NMR (300 MHz, DMSO-*d6*) δ 11.10 (s, 1H), 10.16 (s, 1H), 8.72 (s, 1H), 7.73 (d, *J* = 8.2 Hz, 1H), 7.60 (t, *J* = 7.4 Hz, 2H), 7.00 (d, *J* = 8.2 Hz, 2H), 4.44 (s, 1H), 3.78 (s, 3H), 3.28 (s, 2H), 2.49–2.38 (m, 1H), 1.95 (s, 3H). ^13^C NMR (75 MHz, DMSO-*d6*) δ 166.76, 158.79, 136.97, 135.63, 131.95, 127.47, 126.53, 120.02, 114.43, 59.72, 55.25, 45.78, 29.78, 23.65. HRMS (ESI) *m*/*z* calcd for C_18_H_21_N_2_O_2_ (M + H^+^) 297.1603, found 297.1603.

N-(4′-methoxy-[1,1′-biphenyl]-4-yl)pyrrolidine-3-carboxamide hydrochloride (**2i**). The compound was synthesized according to General procedure C from 4-methoxyphenylboronic acid (0.10 g). Yield 0.095 g (55%), white solid. ^1^H NMR (300 MHz, DMSO-*d6*) δ 10.58 (s, 1H), 9.63 (s, 1H), 9.35 (s, 1H), 7.71 (d, *J* = 8.0 Hz, 2H), 7.57 (d, *J* = 8.0 Hz, 4H), 6.99 (d, *J* = 8.0 Hz, 2H), 3.78 (s, 3H), 3.38–3.14 (m, 5H), 2.35–2.19 (m, 1H), 2.11–1.99 (m, 1H). ^13^C NMR (75 MHz, DMSO-*d6*) δ 170.11, 158.57, 137.68, 134.85, 132.01, 127.26, 126.24, 119.68, 114.29, 55.14, 46.66, 44.69, 43.04, 28.89. HRMS (ESI) *m*/*z* calcd for C_18_H_21_N_2_O_2_ (M + H^+^) 297.1603, found 297.1603.

### 4.3. Antimicrobial Susceptibility Testing

Testing was conducted against the following microorganisms: *Enterococcus faecium* (ATCC 29212), *Staphylococcus aureus* (ATCC 25912), *Klebsiella pneumoniae* (ATCC 19882), *Acinetobacter baumannii* (948^®^, patient-derived strain from the Pasteur Institute own collection), *Pseudomonas aeruginosa* (ATCC 27853), and *Enterobacter cloacae* (ATCC 13047) for compounds **1a**–**g**, **2a**–**i**, and ciprofloxacin (employed as a positive control) using the Kirby–Bauer disk diffusion test [41] under the Standard Operating Procedure of The European Committee on Antimicrobial Susceptibility Testing (EUCAST) [42]. Paper disks bearing 5 mg of the tested compounds were used. Solutions of tested compounds made up in DMSO (1 mg/10 mL) were prepared and diluted to a total volume of 1 mL with deionized water. Aliquots of the resulting solutions (5 µL each) were added to a Petri dish containing Muller–Hilton agar that was inoculated with a bacterial suspension (McFarland OD 1/4 0.5). After the compound solution has dried off, the Petri dish was incubated at 37 °C for 18 h. The bacterial growth inhibition zone diameter around the disk with ciprofloxacin or the compounds’ dried solution circular spot indicated the general susceptibility to a drug being assessed. Thereupon, minimum inhibitory concentrations (MIC, µg/mL) were determined using serial broth dilutions [43]. All measurements were performed in triplicate.

### 4.4. Action Mechanism Evaluation

The antibacterial activity and mechanism of action of the investigated compounds were evaluated using the pDualrep2 reporter system [27] in *E. coli* K12, ΔtolC, and lptD strains transformed with the corresponding plasmid. Overnight cultures were grown in LB medium supplemented with selective antibiotics: 100 μg/mL ampicillin for K12 and ΔtolC strains, and 50 μg/mL kanamycin for the lptD strain. Bacterial cells were plated on Petri dishes containing the respective agar-solidified LB medium with maintenance concentrations of antibiotics, followed by application of 2 μL aliquots of test extracts dissolved in DMSO to a final concentration of 20 mg/mL onto the formed bacterial lawns. Positive controls consisting of erythromycin (5 mg/mL, 1 μL) and ciprofloxacin (2.5 μg/mL, 1 μL) were applied to each plate.

After 24 h incubation at 37 °C, the reporter response was recorded using a ChemiDoc Imaging System (Bio-Rad Laboratories, Hercules, CA, USA). Fluorescence was detected in two spectral channels: Cy5 and Cy3.

### 4.5. Enzyme Interaction Assays

The following assay mixes were used to study the interaction of target substances with enzymes:

(1) 3× Gyrase assay mix: 105 mM Tris-HCl, pH 7.5, 18 mM MgCl_2_, 5.4 mM spermidine, 72 mM KCl, 15 mM DTT, 1.08 mg/mL BSA, 19.5% glycerol (*w*/*v*) [44];
(2) 5× Topo4 assay mix: 250 mM Tris-HCl, pH 7.5, 500 mM potassium glutamate, 50 mM magnesium acetate, 50 mM DTT, 50 μg/mL BSA [45];
(3) 5× dye mix: 5% SDS, 25% glycerol, 0.25 mg/mL bromophenol blue.

Gyrase (GyrA and GyrB subunits), topoisomerase IV (ParC and ParE subunits), topoisomerase I (TopA) enzymes (obtained and purified according to the reported procedure [46]), and Klenow fragment (New England Biolabs, Ipswich, MA, USA) were used in tests. The tests also utilized relaxed or supercoiled pHot plasmid substrate (TopoGEN, Buena Vista, CO, USA), cDNA (Inspiralis, Norwich, UK), proteinase K, agarose gel (1% in TBE), 10 mg/mL ethidium bromide solution, and 25 mM ATP solution.

#### 4.5.1. Klenow Fragment Polymerization Test

The first cocktail, containing 0.5 μL of 10× Klenow fragment buffer, 0.5 μL of dNTPs (2 mM), 1 μL of a mixture of primers (Table S3), and 3 μL of sterile water, was being prepared on ice. Then, the cocktail was incubated for 5 min at 95 °C and slowly cooled to room temperature. A second cocktail, containing 0.5 μL of 10× Klenow fragment buffer, 0.25 μL of Klenow fragment, 2 μL of testing compound, and 2.25 μL of sterile water, was being prepared on ice. Then, both cocktails were mixed and incubated for 30 min at 37 °C.

Ten percent of PAAG was cast and used for electrophoresis in denaturing conditions in a TBE buffer, first for 10 min at 80 V and then at 160 V for 1 h. The resulting gel was stained for 15 min in a bath with an equal volume of 2× intercalating dye solution (0.2 mg/mL ethidium bromide) and then scanned on a Typhoon^tm^ laser scanner (Cytiva, Marlborough, MA, USA).

#### 4.5.2. Topoisomerase I Relaxation Test

A mixture, containing 2 μL of 3× Gyrase assay mix, 0.33 μL of supercoiled DNA (i.e., 100 ng), 1 μL of testing compound in DMSO (1 mg/10 mL), and 1.67 μL of sterile water, was made up on ice. 1 μL of topoisomerase I was added last. The reactants were mixed and incubated for 1 h at 37 °C. The reaction was stopped by adding 4 μL of 5× dye mix. The DNA products were separated by electrophoresis on a 1% agarose gel run overnight at 16 V. The gel was stained in TBE containing 0.5 μg/mL EtBr for 30 min and scanned on a Typhoon™ laser scanner.

#### 4.5.3. Topoisomerase IV Relaxation Cleavage Test

A mixture, containing 1.2 μL of 5× Topo4 assay mix, 0.33 μL of supercoiled DNA (i.e., 100 ng), 1 μL of testing compound solution, and 2.5 μL of sterile water, was made up on ice. 1 μL of topoisomerase IV was added last. The reactants were mixed and incubated for 1 h at 37 °C. DNA cleavage was induced by adding 1 μL of 2% (*w*/*v*) SDS and 1 μL of 1 mg/mL proteinase K. These solutions were mixed and incubated for 30 min at 37 °C. The reaction was stopped by adding 5 μL of 5× dye mix. Electrophoretic analysis of DNA products was carried out as in Section 4.5.2.

#### 4.5.4. Topoisomerase IV Decatenation Cleavage Test

A mixture containing 1.2 μL of 5× Topo4 assay mix, 1 μL of cDNA (i.e., 100 ng), 1 μL of ATP (25 mM), 1 μL of testing compound, and 0.8 μL of sterile water was made up on ice. Lastly, 1 μL of topoisomerase IV was added. The reactants were mixed and incubated for 1 h at 37 °C. DNA cleavage was induced by adding 1 μL of 2% (*w*/*v*) SDS and 1 μL of 1 mg/mL proteinase K. These solutions were mixed and incubated for 30 min at 37 °C. The reaction was stopped by adding 5 μL of 5× dye mix. Electrophoretic analysis of DNA products was carried out as in Section 4.5.2.

#### 4.5.5. DNA Gyrase Relaxation Cleavage Test

A mixture, containing 2 μL of 3× gyrase assay mix, 0.33 μL of supercoiled DNA (i.e., 100 ng), 1 μL of testing compound, and 1.67 μL of sterile water, was made up on ice. Lastly, 1 μL of DNA gyrase was added. The reactants were mixed and incubated for 1 h at 37 °C. DNA cleavage was induced by adding 1 μL of 2% (*w*/*v*) SDS and 1 μL of 1 mg/mL proteinase K. These solutions were mixed and incubated for 30 min at 37 °C. The reaction was stopped by adding 5 μL of 5× dye mix. Electrophoretic analysis of DNA products was carried out as in Section 4.5.2.

#### 4.5.6. DNA Gyrase Supercoiling Cleavage Test

A mixture, containing 2 μL of 3× gyrase assay mix, 1 μL of relaxed DNA (i.e., 100 ng), 0.2 μL of ATP (25 mM), 1 μL of testing compound, and 0.8 μL of sterile water, was made up on ice. 1 μL of DNA gyrase was added last. The reactants were mixed and incubated for 1 h at 37 °C. DNA cleavage was induced by adding 1 μL of 2% (*w*/*v*) SDS and 1 μL of 1 mg/mL proteinase K. These solutions were mixed and incubated for 30 min at 37 °C. The reaction was stopped by adding 5 μL of 5× dye mix. The analysis of DNA products was carried out as in Section 4.5.2.

### 4.6. MTT Assay

Cytotoxicity was assessed using the MTT (3-(4,5-dimethylthiazol-2-yl)2,5-diphenyl tetrazolium bromide) assay [47], with some modifications.

5000 cells per well for VA13 and MCF7 and 2500 cells per well for HEK293T, HCT116, PC3, and A549 cell lines were plated out in 140 μL of DMEM/F12 media (PanEco LLC, Moscow, Russia) in a 96-well plate and incubated at 37 °C and 5% CO_2_ for 18 h before treatment. Substances were dissolved in DMSO. Tested samples were suspended in cell growth media, and 11 μL of dilutions were added to cells; the dilution step was ×3. Pure DMSO solution was used as a control of the solvent’s toxicity. Maximum DMSO concentration in the cell growth media after adding the substances was less than 1%. After adding the samples, the cells were incubated for 72 h, then metabolic activity was measured with the MTT test. MTT reagent (Paneco LLC, Moscow, Russia) was added into the media up to 0.5 mg/mL. Cells were incubated with MTT for 2–4 h, then the media was removed and displaced with 100 μL of DMSO. The amount of MTT reduced by cells to its blue formazan derivative was measured spectrophotometrically at 565 nM using a VICTOR microplate reader (PerkinElmer, Singapore) and normalized to the values for cells incubated without compounds.

### 4.7. Molecular Modeling

#### 4.7.1. Protein Preparation

All proteins were downloaded from RCSB Protein Data Bank (accesion numbers 6OZW, 9N26, 5NPK, 7MVS, 4K4O, 8BN6/7PTF, and 7PQL) and preprocessed before calculations using the protein prepwizard tool from the Schrodinger suite. During preprocessing, errors such as missing amino acid sidechains and loops, incorrect protonation states, missing hydrogens, incorrect bond orders, angles, etc., were fixed. Solvent molecules (waters) were deleted from protein models, and restrained minimization was performed (for XRAY structures) [48]. The three-dimensional ligand geometry is generated by the LigPrep module. All molecular modeling operations are carried out in the OPLS4 force field [49].

#### 4.7.2. Induced-Fit Docking of Molecules

Docking grid boxes for all observed proteins are calculated based on reference ligand position and size (grid placement on complex ligand centroid; maximum grid side is 12 Å). In the case of ligand absence, grid boxes are calculated by residues involved in potential interactions. For each ligand, 20 solutions were generated. The best-fitting pose was selected by comparison with reference ligand positioning in the protein active site if it is present in the PDB files.

The binding pose clustering is used as a key parameter, showing ligand binding quality. Additionally, the observed cluster must replicate the pharmacophore characteristics of the reference ligands (if present). This value is shown as stars in parentheses. Three stars mean more than 60% clustered docking solutions with RMSD less than 1.5 Å, two stars mean 40–60% clustered docking solutions with RMSD less than 3 Å, and one star means less than 40% or solutions with no clustering.

## 5. Conclusions

In conclusion, this work has successfully yielded a new class of potent antibacterial agents, with several analogs exhibiting a 10- to 100-fold increase in activity against ESKAPE pathogens compared to ciprofloxacin. Preliminary SAR analysis, while limited in scope, showed lipophilicity of the aromatic ring A and the presence of para-halogen substituents as factors for enhancing antimicrobial potency. Mechanistic studies indicate that these compounds likely exert their effect by disrupting nucleic acid replication, as evidenced by their inhibition of DNA gyrase and topoisomerase I, rather than through protein biosynthesis. However, this promising antibacterial profile is accompanied by significant cytotoxicity, consistent with a mechanism involving DNA intercalation. Despite this, the selectivity index for the most active compounds suggests a window that can be exploited for further optimization.

It should be noted that a new chemotype for antibacterials has been presented. Further optimization should unlock the potential of this new core for the broad-spectrum antibiotics design.

## Figures and Tables

**Table 1:** Antibacterial activity as a minimal inhibitory concentration (MIC, µg/mL) of compounds **1a**–**g**, **2a**–**i**, and ciprofloxacin (positive control) against the ESKAPE panel of pathogens; nt—not tested. The MIC values are the mean from three different assays (errors were in the range of ±5–10% of the reported values). The compounds considered in the in silico studies are highlighted by green.

Species	Compound												
	1a	1b	1d	1e	1f	1g	2b	2c	2f	2g	2h	2i	Cyp
*E. faecium*	nt	0.16	0.65	0.09	0.19	0.20	0.10	2.6	0.17	0.36	0.40	0.35	1.25
*S. aureus*	0.20	0.16	0.17	0.09	0.19	0.10	0.10	0.08	0.08	0.18	0.20	0.18	1.25
*K. pneumoniae*	0.20	0.16	0.33	0.09	0.19	0.10	0.10	0.08	0.34	0.36	0.80	0.35	0.6
*A. baumannii*	0.20	0.65	nt	0.09	0.19	0.10	0.10	0.08	0.08	0.36	0.20	0.70	2.5
*P. aeruginosa*	nt	0.33	2.6	0.09	0.19	0.81	0.79	0.08	0.64	0.18	3.2	0.70	0.6
*E. cloacae*	0.20	0.16	0.17	0.09	0.19	0.20	0.39	0.08	0.34	0.18	0.40	0.18	3.0

**Table 2:** Results of molecular docking of the selected compounds into the active sites of microbial targets.

Compound Docking Score (kcal/mol) and Binding Pose Quality (Stars)					
**topA**	**2c**	**1b**	**1e**	**2d**	**2g**
*Staphylococcus aureus*	−4.93 *	−5.12 *	−5.19 *	−5.00 *	−5.17 ***
**GyrA/B**					
*Enterococcus faecium*	−5.70 *	−5.43 *	−5.71 ***	−4.60 **	−5.89 *
*Staphylococcus aureus*	−5.0 *	−4.02 *	−5.34 *	−6.39 ***	−6.53 ***
*Acinetobacter Baumannii*	−5.56 **	−4.01 *	−4.74 **	−5.00 ***	−5.54 ***
*Pseudomonas aeruginosa*	−5.67 **	−5.23 *	−4.67 *	−5.70 ***	−5.15 **
**Pbp2a**					
*Staphylococcus aureus*	−4.24 **	−5.78 **	−6.18 ***	−4.98 **	−4.35 *

Stars indicate the quality of binding pose, *** is good, ** is fair, * is low.

## Data Availability

The original contributions presented in this study are included in the article/Supplementary Materials. Further inquiries can be directed to the corresponding author.

## Supplementary Materials

The following supporting information can be downloaded at: https://www.mdpi.com/article/10.3390/molecules31020240/s1.

## Abbreviations

The following abbreviations are used in this manuscript:

ESKAPE	acronym comprising the scientific names of six bacterial pathogens
MIC	minimum inhibitory concentrations
AI	artificial intelligence
MRSA	multiresistant strains of *S. aureus*
SAR	structure–activity relation
HBTU	hexafluorophosphate benzotriazole tetramethyl uronium
TopA	topoisomerase I
IC50abs	half maximal inhibitory concentration
PDB	Protein Data Bank
RMSD	root mean square deviation of atomic positions

## References

[1] Darby E.M., Trampari E., Siasat P., Gaya M.S., Alav I., Webber M.A., Blair J.M.A. (2023). Molecular mechanisms of antibiotic resistance revisited. Nat. Rev. Microbiol..

[2] Brüssow H. (2024). The antibiotic resistance crisis and the development of new antibiotics. Microb. Biotechnol..

[3] de la Fuente-Nunez C., Cesaro A., Hancock R.E.W. (2023). Antibiotic failure: Beyond antimicrobial resistance. Drug Resist. Updates.

[4] Farha M.A., Tu M.M., Brown E.D. (2025). Important challenges to finding new leads for new antibiotics. Curr. Opin. Microbiol..

[5] Butler M.S., Vollmer W., Goodall E.C.A., Capon R.J., Henderson I.R., Blaskovich M.A.T. (2024). A Review of Antibacterial Candidates with New Modes of Action. ACS Infect. Dis..

[6] Bergkessel M., Forte B., Gilbert I.H. (2023). Small-Molecule Antibiotic Drug Development: Need and Challenges. ACS Infect. Dis..

[7] Nandikolla A., Khetmalis Y.M., Sangeetha G.P.V., Chandu A., Swati, Kumar M.M.K., Sharma V., Murugesan S., Chandra Sekhar K.V.G. (2023). Tetrahydroisoquinoline based 5-nitro-2-furoic acid derivatives: A promising new approach for anti-tubercular agents. New J. Chem..

[8] Xie Y.P., Sangaraiah N., Meng J.P., Zhou C.H. (2022). Unique Carbazole-Oxadiazole Derivatives as New Potential Antibiotics for Combating Gram-Positive and -Negative Bacteria. J. Med. Chem..

[9] Lukin A., Chudinov M., Vedekhina T., Rogacheva E., Kraeva L., Bakulina O., Krasavin M. (2022). Exploration of Spirocyclic Derivatives of Ciprofloxacin as Antibacterial Agents. Molecules.

[10] Bian X., Qu X., Zhang J., Nang S.C., Bergen P.J., Tony Zhou Q., Chan H.K., Feng M., Li J. (2022). Pharmacokinetics and pharmacodynamics of peptide antibiotics. Adv. Drug Deliv. Rev..

[11] Bodrova T.G., Budanova U.A., Sebyakin Y.L. (2024). L-Ornithine derivatives with structural hetaryl and alkyl moiety: Synthesis and antibacterial activity. Fine Chem. Technol..

[12] Chang R.Y.K., Nang S.C., Chan H.K., Li J. (2022). Novel antimicrobial agents for combating antibiotic-resistant bacteria. Adv. Drug Deliv. Rev..

[13] Kong Q., Yang Y. (2021). Recent advances in antibacterial agents. Bioorg Med. Chem. Lett..

[14] Mitcheltree M.J., Pisipati A., Syroegin E.A., Silvestre K.J., Klepacki D., Mason J.D., Terwilliger D.W., Testolin G., Pote A.R., Wu K.J.Y. (2021). A synthetic antibiotic class overcoming bacterial multidrug resistance. Nature.

[15] Liu G., Catacutan D.B., Rathod K., Swanson K., Jin W., Mohammed J.C., Chiappino-Pepe A., Syed S.A., Fragis M., Rachwalski K. (2023). Deep learning-guided discovery of an antibiotic targeting *Acinetobacter baumannii*. Nat. Chem. Biol..

[16] Stokes J.M., Yang K., Swanson K., Jin W., Cubillos-Ruiz A., Donghia N.M., MacNair C.R., French S., Carfrae L.A., Bloom-Ackermann Z. (2020). A Deep Learning Approach to Antibiotic Discovery. Cell.

[17] Lluka T., Stokes J.M. (2023). Antibiotic discovery in the artificial intelligence era. Ann. N. Y. Acad. Sci..

[18] Spink E., Ding D., Peng Z., Boudreau M.A., Leemans E., Lastochkin E., Song W., Lichtenwalter K., O’Daniel P.I., Testero S.A. (2015). Structure-activity relationship for the oxadiazole class of antibiotics. J. Med. Chem..

[19] O’Daniel P.I., Peng Z., Pi H., Testero S.A., Ding D., Spink E., Leemans E., Boudreau M.A., Yamaguchi T., Schroeder V.A. (2014). Discovery of a new class of non-beta-lactam inhibitors of penicillin-binding proteins with Gram-positive antibacterial activity. J. Am. Chem. Soc..

[20] Buommino E., De Marino S., Sciarretta M., Piccolo M., Festa C., D’Auria M.V. (2021). Synergism of a Novel 1,2,4-oxadiazole-containing Derivative with Oxacillin against Methicillin-Resistant *Staphylococcus aureus*. Antibiotics.

[21] Naclerio G.A., Abutaleb N.S., Onyedibe K.I., Karanja C., Eldesouky H.E., Liang H.W., Dieterly A., Aryal U.K., Lyle T., Seleem M.N. (2022). Mechanistic Studies and In Vivo Efficacy of an Oxadiazole-Containing Antibiotic. J. Med. Chem..

[22] Qin H.L., Liu J., Fang W.Y., Ravindar L., Rakesh K.P. (2020). Indole-based derivatives as potential antibacterial activity against methicillin-resistance *Staphylococcus aureus* (MRSA). Eur. J. Med. Chem..

[23] Qian Y., Birhanu B.T., Yang J., Ding D., Janardhanan J., Mobashery S., Chang M. (2023). A Potent and Narrow-Spectrum Antibacterial against *Clostridioides difficile* Infection. J. Med. Chem..

[24] Vinogradova L.V., Komarova K.Y., Chudinov M.V., Rogacheva E.V., Kraeva L.A., Lukin A.Y. (2024). Scaffold hopping in the oxadiazole antibiotic structure leads to more active compounds. Mendeleev Commun..

[25] Boudreau M.A., Ding D., Meisel J.E., Janardhanan J., Spink E., Peng Z., Qian Y., Yamaguchi T., Testero S.A., O’Daniel P.I. (2020). Structure-Activity Relationship for the Oxadiazole Class of Antibacterials. ACS Med. Chem. Lett..

[26] Pendleton J.N., Gorman S.P., Gilmore B.F. (2013). Clinical relevance of the ESKAPE pathogens. Expert Rev. Anti-Infect. Ther..

[27] Osterman I.A., Komarova E.S., Shiryaev D.I., Korniltsev I.A., Khven I.M., Lukyanov D.A., Tashlitsky V.N., Serebryakova M.V., Efremenkova O.V., Ivanenkov Y.A. (2016). Sorting Out Antibiotics’ Mechanisms of Action: A Double Fluorescent Protein Reporter for High-Throughput Screening of Ribosome and DNA Biosynthesis Inhibitors. Antimicrob. Agents Chemother..

[28] Baba T., Ara T., Hasegawa M., Takai Y., Okumura Y., Baba M., Datsenko K.A., Tomita M., Wanner B.L., Mori H. (2006). Construction of *Escherichia coli* K-12 in-frame, single-gene knockout mutants: The Keio collection. Mol. Syst. Biol..

[29] Orelle C., Carlson S., Kaushal B., Almutairi M.M., Liu H., Ochabowicz A., Quan S., Pham V.C., Squires C.L., Murphy B.T. (2013). Tools for characterizing bacterial protein synthesis inhibitors. Antimicrob. Agents Chemother..

[30] Lim D.C., Strynadka N.C.J. (2002). Structure of penicillin G acyl-Penicillin binding protein 2a from methicillin resistant *Staphylococcus aureus* strain 27r at 2.45 A resolution. Nat. Struct. Biol..

[31] Verma S.K., Verma R., Kumar K.S.S., Banjare L., Shaik A.B., Bhandare R.R., Rakesh K.P., Rangappa K.S. (2021). A key review on oxadiazole analogs as potential methicillin-resistant *Staphylococcus aureus* (MRSA) activity: Structure-activity relationship studies. Eur. J. Med. Chem..

[32] Singh S., Geetha P., Ramajayam R. (2023). Isolation, synthesis and medicinal chemistry of biphenyl analogs—A review. Results Chem..

[33] Liu B., Wang Y., Chen N., Li C., Zhao J., Li T. (2024). Diphenyl Ethers: Isolation, Bioactivities and Biosynthesis. Mini-Rev. Org. Chem..

[34] Ghouse S.M., Akhir A., Sinha K., Pawar G., Saxena D., Akunuri R., Malik P., Roy A., Parida K.K., Dasgupta A. (2023). Synthesis and Biological Evaluation of Diphenyl Ether-Linked Quinolone Derivatives as Antibacterial Agents. ChemistrySelect.

[35] Wang D., Wang C., Gui P., Liu H., Khalaf S.M.H., Elsayed E.A., Wadaan M.A.M., Hozzein W.N., Zhu W. (2017). Identification, Bioactivity, and Productivity of Actinomycins from the Marine-Derived *Streptomyces heliomycini*. Front. Microbiol..

[36] Wadia S., Tran B. (2014). Colistin-mediated neurotoxicity. BMJ Case Rep..

[37] Giacobbe D.R., di Masi A., Leboffe L., Del Bono V., Rossi M., Cappiello D., Coppo E., Marchese A., Casulli A., Signori A. (2018). Hypoalbuminemia as a predictor of acute kidney injury during colistin treatment. Sci. Rep..

[38] Tegge W., Guerra G., Holtke A., Schiller L., Beutling U., Harmrolfs K., Grobe L., Wullenkord H., Xu C., Weich H. (2021). Selective Bacterial Targeting and Infection-Triggered Release of Antibiotic Colistin Conjugates. Angew. Chem. Int. Ed..

[39] Oh S., Kwon D.Y., Choi I., Kim Y.M., Lee J.Y., Ryu J., Jeong H., Kim M.J., Song R. (2021). Identification of Piperidine-3-carboxamide Derivatives Inducing Senescence-like Phenotype with Antimelanoma Activities. ACS Med. Chem. Lett..

[40] Benamara N., Merabet-Khelassi M., Aribi-Zouioueche L., Riant O. (2021). CAL-B-mediated efficient synthesis of a set of valuable amides by direct amidation of phenoxy- and aryl-propionic acids. Chem. Pap..

[41] Bauer A.W., Kirby W.M.M., Sherris J.C., Turck M. (1966). Antibiotic Susceptibility Testing by a Standardized Single Disk Method. Am. J. Clin. Pathol..

[42] EUCAST SOP 9.2. Procedure for Establishing Zone Diameter Breakpoints and Quality Control Criteria. https://www.eucast.org/fileadmin/src/media/PDFs/EUCAST_files/EUCAST_SOPs/2020/EUCAST_SOP_9.2_Disk_diffusion_breakpoints_and_QC_ranges_final.pdf.

[43] Wiegand I., Hilpert K., Hancock R.E. (2008). Agar and broth dilution methods to determine the minimal inhibitory concentration (MIC) of antimicrobial substances. Nat. Protoc..

[44] Fisher L.M., Pan X.S. (2008). Methods to assay inhibitors of DNA gyrase and topoisomerase IV activities. Methods Mol. Med..

[45] Peng H., Marians K.J. (1999). Overexpression and purification of bacterial topoisomerase IV. Methods Mol. Biol..

[46] Shiriaev D.I., Sofronova A.A., Berdnikovich E.A., Lukianov D.A., Komarova E.S., Marina V.I., Zakalyukina Y.V., Biryukov M.V., Maviza T.P., Ivanenkov Y.A. (2023). Nybomycin inhibits both types of *E. coli* DNA gyrase-fluoroquinolone-sensitive and fluoroquinolone-resistant. Antimicrob. Agents Chemother..

[47] Mosmann T. (1983). Rapid colorimetric assay for cellular growth and survival: Application to proliferation and cytotoxicity assays. J. Immunol. Methods.

[48] Sastry G.M., Adzhigirey M., Day T., Annabhimoju R., Sherman W. (2013). Protein and ligand preparation: Parameters, protocols, and influence on virtual screening enrichments. J. Comput.-Aided Mol. Des..

[49] Lu C., Wu C., Ghoreishi D., Chen W., Wang L., Damm W., Ross G.A., Dahlgren M.K., Russell E., Von Bargen C.D. (2021). OPLS4: Improving Force Field Accuracy on Challenging Regimes of Chemical Space. J. Chem. Theory Comput..

