# Supplementary material for: Scaffold Simplification Yields Potent Antibacterial Agents That Target Bacterial Topoisomerases
Source: Molecules. 2026 Jan 10;31(2):240. doi: 10.3390/molecules31020240 (PMC12844012; doi:10.3390/molecules31020240)

Supporting Information

Scaffold simplification yields potent antibacterial agents that target bacterial topoisomerases

Lyubov Khudiakova [1], Kristina Komarova [1], Maxim Zhuravlev [1], Dmitry Deniskin [1], Alexey Golovanov [1], Artemiy Nichugovskiy [1], Kirill Babkin [1], Maria Zakharova [1], Mikhail Chudinov [1,*], Elizaveta Rogacheva [2], Lyudmila Kraeva [2], Olga Shevtsova [3], Daria Ipatova [3], Dmitry Skvortsov [3], Dmitrii Lukianov [3,4], Maxim Kryakvin [4], Maxim Gureev [5] and Alexey Lukin [6]

1 Lomonosov Institute of Fine Chemical Technologies, MIREA—Russian Technological University, Moscow 119454, Russia
2 Pasteur Institute of Epidemiology and Microbiology, Saint Petersburg 197101, Russia
3 Department of Chemistry, Lomonosov Moscow State University, GSP-1, 1-3 Leninskiye Gory, Moscow 119991, Russia
4 Center for Molecular and Cellular Biology, 121205 Moscow, Russia
5 Laboratory of Bio- and Cheminformatics, School of Computer Science, Physics and Technology, HSE University, 190121, Saint Petersburg, Russia
6 Department of Medical Chemistry, Institute of Chemistry, Saint Petersburg State University, Saint Petersburg 199034, Russia
* Correspondence: chudinov@mirea.ru

Tables S1-S3. 3

Table S1. Antibacterial activity as a bacterial growth inhibition zone diameter (IZ, mm) of compounds 1a–g, 2a–i and ciprofloxacin (positive control) against the ESKAPE panel of pathogens. 3

Table S2. Cytotoxicity of the evaluated compounds by the MTT test. 3

Table S3. Primers used in the Klenow fragment polymerisation assay and the resulting product. The overlap area is highlighted in bold. 3

NMR spectra of synthesized compounds 4

^{1}H and ^{13}C NMR spectra for compound 1a 4

^{1}H and ^{13}C NMR spectra for compound 1b 5

^{1}H and ^{13}C NMR spectra for compound 1c 6

^{1}H and ^{13}C NMR spectra for compound 1d 7

^{1}H and ^{13}C NMR spectra for compound 1e 8

^{1}H and ^{13}C NMR spectra for compound 1f 9

^{1}H and ^{13}C NMR spectra for compound 1g 10

^{1}H and ^{13}C NMR spectra for compound 2a 11

^{1}H and ^{13}C NMR spectra for compound 2b 12

^{1}H and ^{13}C NMR spectra for compound 2c 13

^{1}H and ^{13}C NMR spectra for compound 2d 14

^{1}H and ^{13}C NMR spectra for compound 2e 15

^{1}H and ^{13}C NMR spectra for compound 2f 16

^{1}H and ^{13}C NMR spectra for compound 2g 17

^{1}H and ^{13}C NMR spectra for compound 2h 18

^{1}H and ^{13}C NMR spectra for compound 2i 19

^{1}H and ^{13}C NMR spectra for compound 5a 20

^{1}H and ^{13}C NMR spectra for compound 5b 21

^{1}H and ^{13}C NMR spectra for compound 5c 22

^{1}H and ^{13}C NMR spectra for compound 5d 23

Tables S1-S3.

Table S1. Antibacterial activity as a bacterial growth inhibition zone diameter (IZ, mm) of compounds 1a–g, 2a–i and ciprofloxacin (positive control) against the ESKAPE panel of pathogens. The IZ values are the mean from three different assays (errors were in the range of ± 5 –10% of the reported values).

**Species**	**Compound**																
	**1a**	**1b**	**1c**	**1d**	**1e**	**1f**	**1g**	**2a**	**2b**	**2c**	**2d**	**2e**	**2f**	**2g**	**2h**	**2i**	**Cyp**
E.faecium	0	11	0	13	5	7	13	11	10	10	0	0	9	9	7	9	17
S.aureus	15	22	0	7	0.09	14	11	0	17	14	0	0	12	11	12	11	21
K.pneumoniae	19	19	0	9	0.09	13	12	7	16	14	0	0	10	18	9	9	27
A.baumannii	11	9	13	0	0.09	9	10	0	11	16	0	0	6	10	9	8	14
P.aeruginosa	0	11	0	9	0.09	8	13	0	9	11	0	0	7	9	9	6	25
E. cloacae	9	9	0	5	0.09	7	11	0	9	13	0	0	7	11	7	7	21

Table S2. Cytotoxicity of the evaluated compounds by the MTT test.

**Compound**	IC_{50abs} (μg/mL) **Cell line**				
	**HEK293T**	**MCF7**	**VA13**	**HCT116**	**PC3**
**2c**	4,2±0,3	8±1	3,5±0,6	1,9±0,4	4,7±0,5
**2b**	2,4±0,4	8,0±0,7	2,3±0,3	1,8±0,4	3,1±0,2

Table S3. Primers used in the Klenow fragment polymerisation assay and the resulting product. The overlap area is highlighted in bold.

**Name**	**Sequence**	**Length, nt**
RYY_tp_TSR	GGTTATAATGAATTTTGCTTATTAACGATAGAATTCTATCACATTCTTGATTC**TTAACTACGACCA CAATTACG**	74
RYY_tp	AAGTATAAGGAGGAAAACATATGTGTCATCGTGATTATCGTTATTATAATGGTTATGGTGGTTGTG GTC**CGTAATTGTGGTCGTAGTTAA**	89
**Product**	GGTTATAATGAATTTTGCTTATTAACGATAGAATTCTATCACATTCTTGATTC**TTAACTACGACCA CAATTACG**ACCACAACCACCATAACCATTATAATAACGATAATCACGATGACACATATGTTTTCC TCCTTATACTT	142

NMR spectra of synthesized compounds

^{1}H and ^{13}C NMR spectra for compound 1a

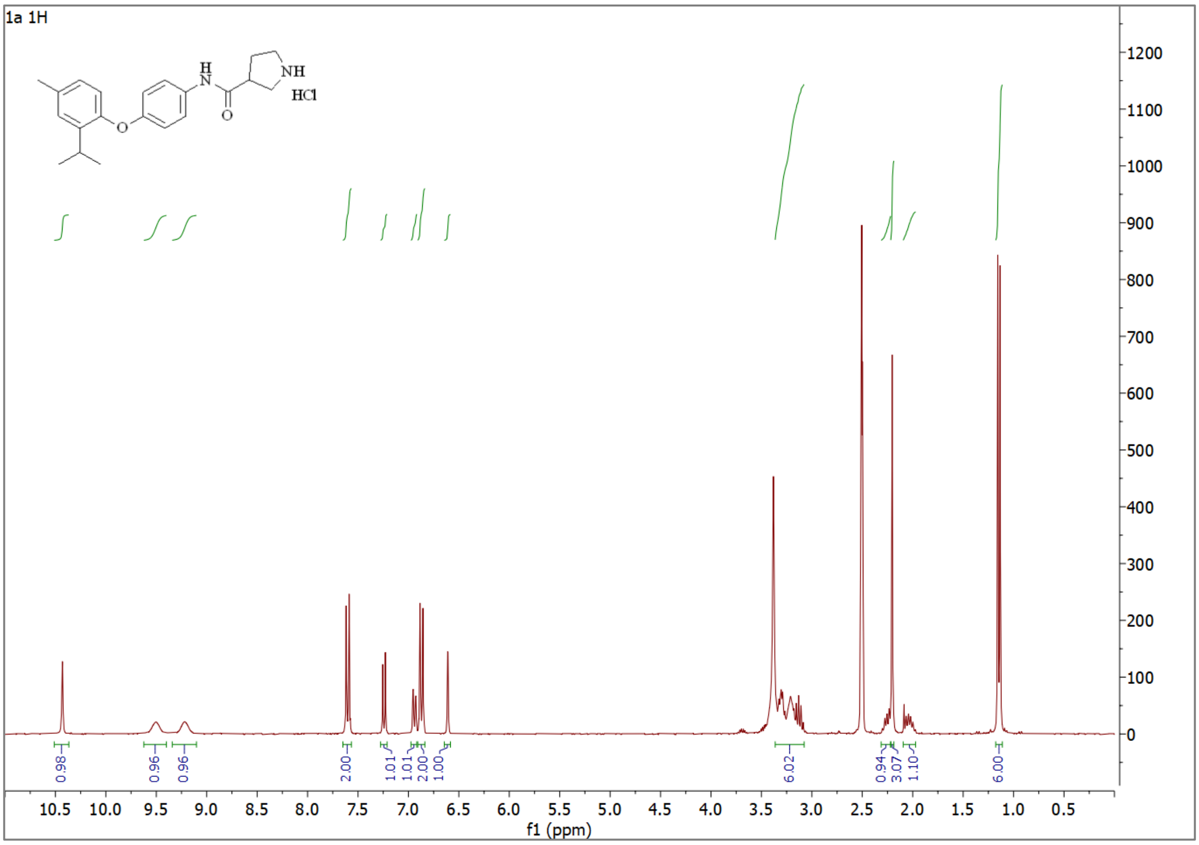

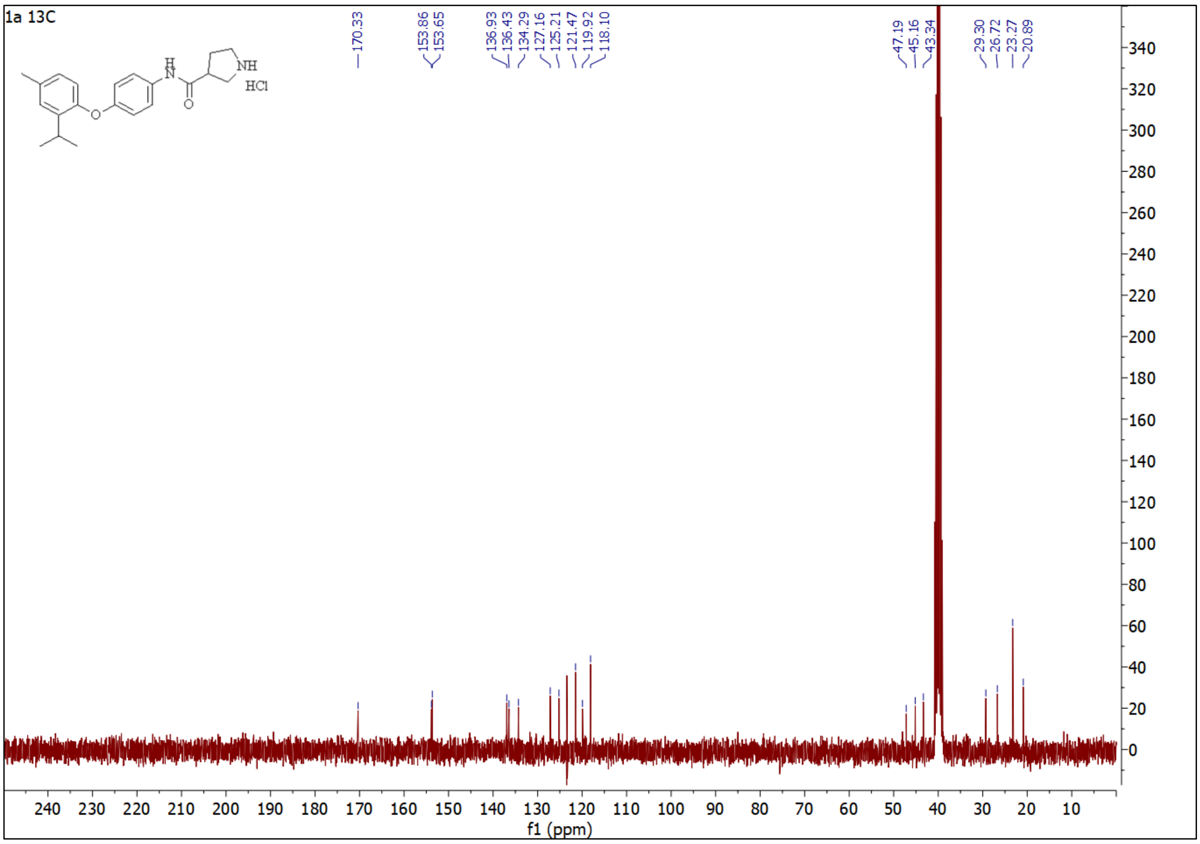

^{1}H and ^{13}C NMR spectra for compound 1b

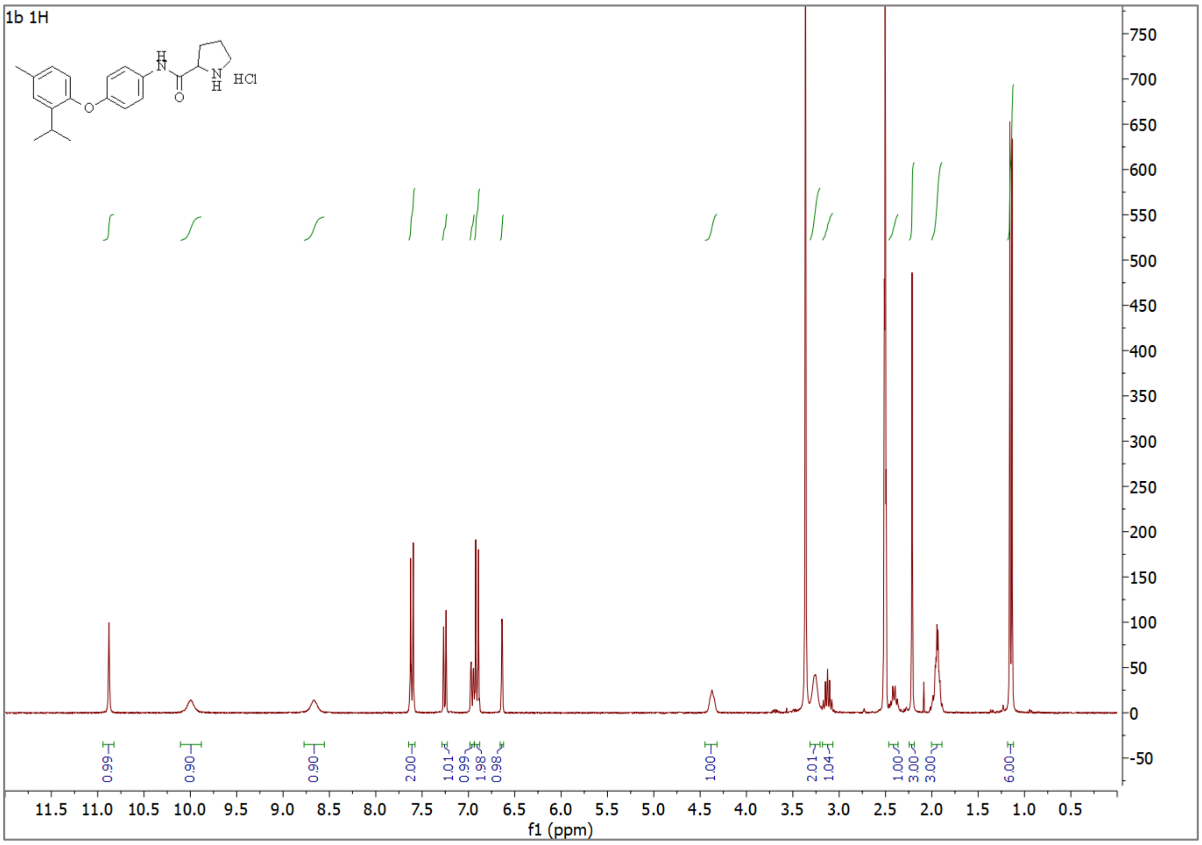

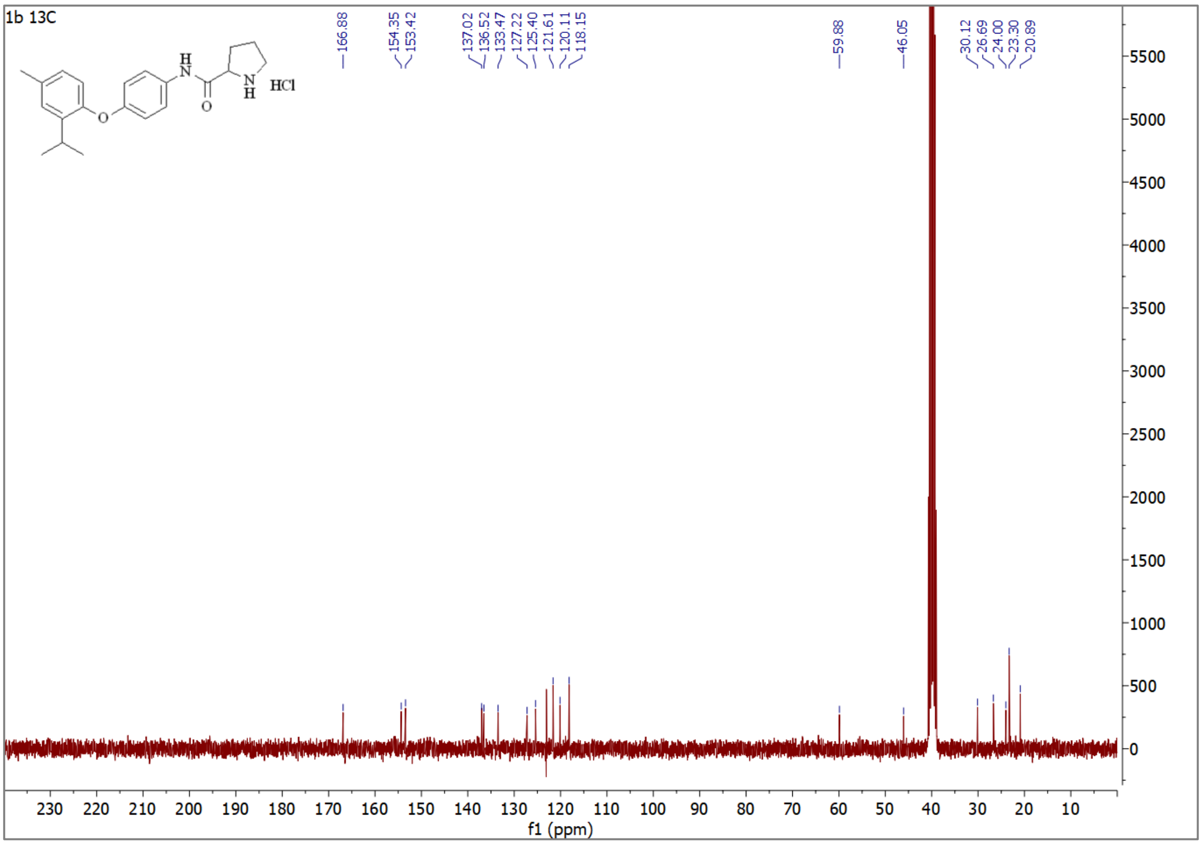

^{1}H and ^{13}C NMR spectra for compound 1c

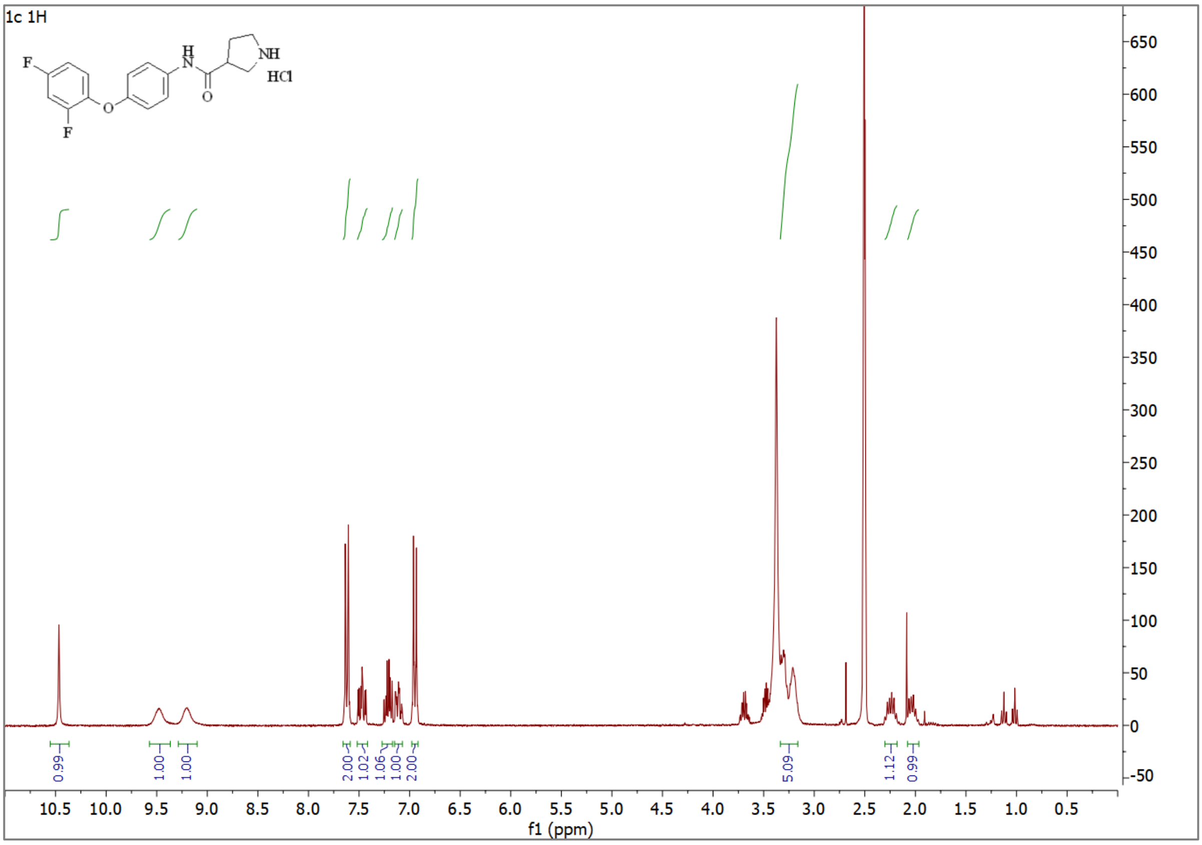

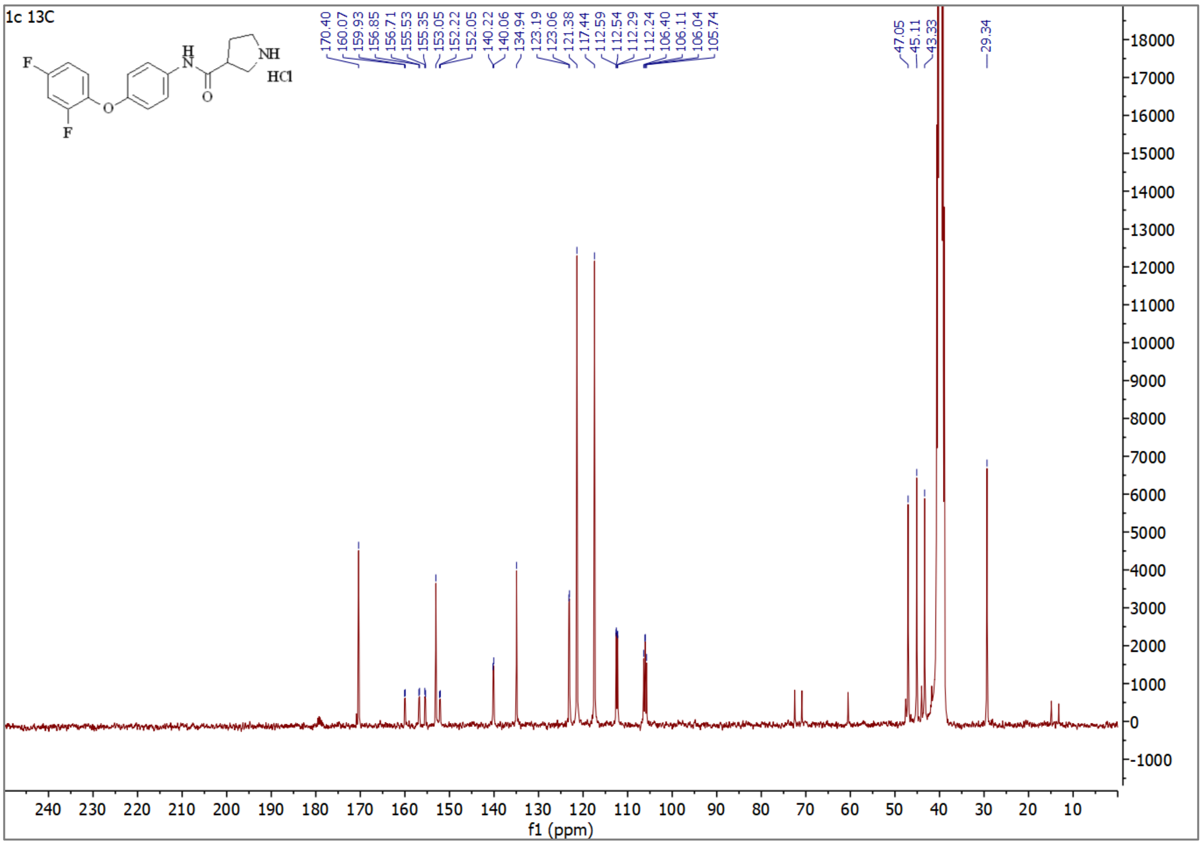

^{1}H and ^{13}C NMR spectra for compound 1d

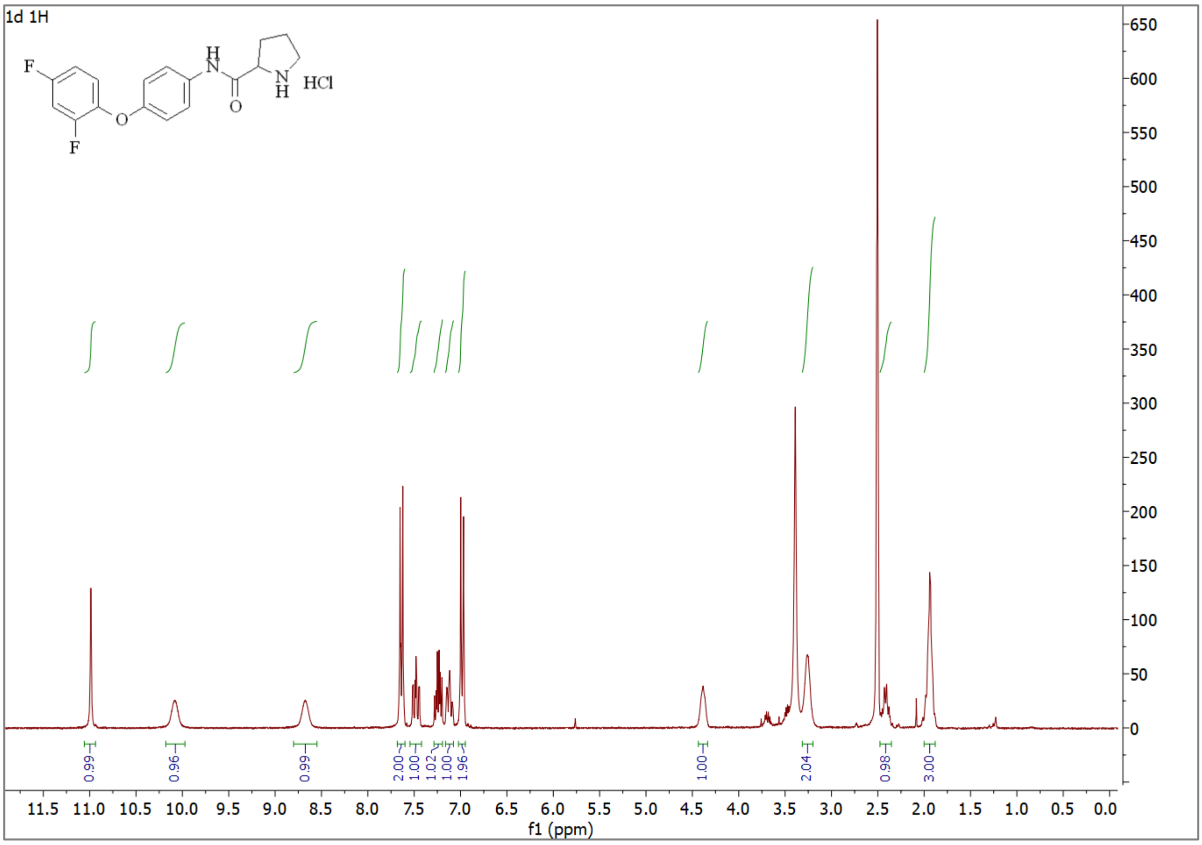

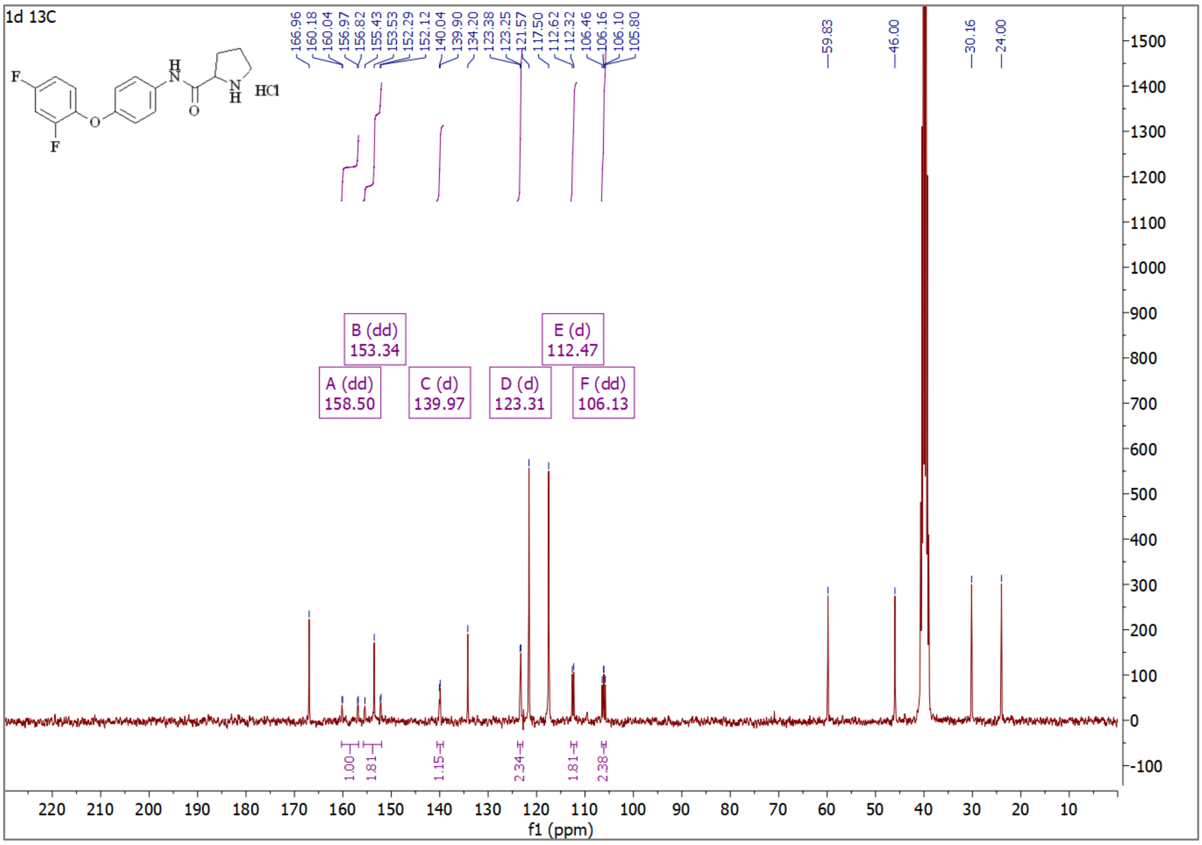

^{1}H and ^{13}C NMR spectra for compound 1e

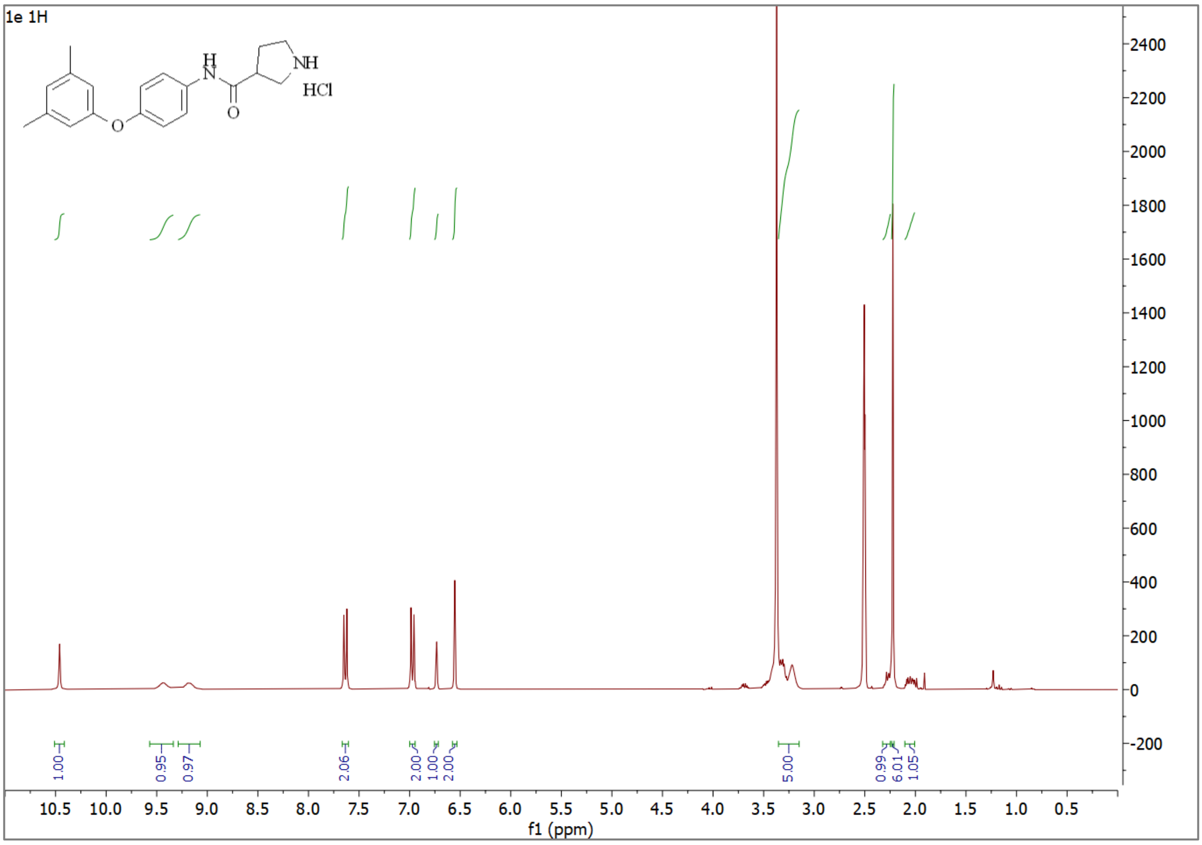

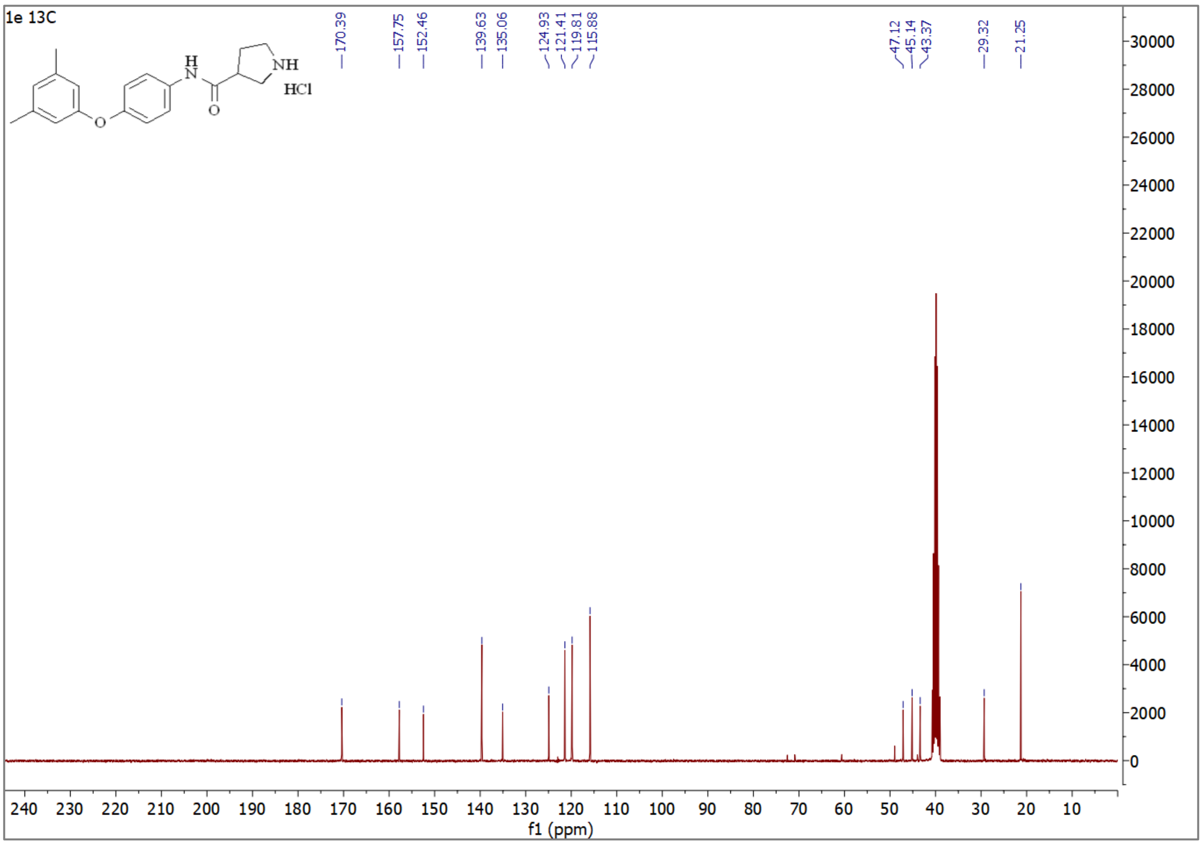

^{1}H and ^{13}C NMR spectra for compound 1f

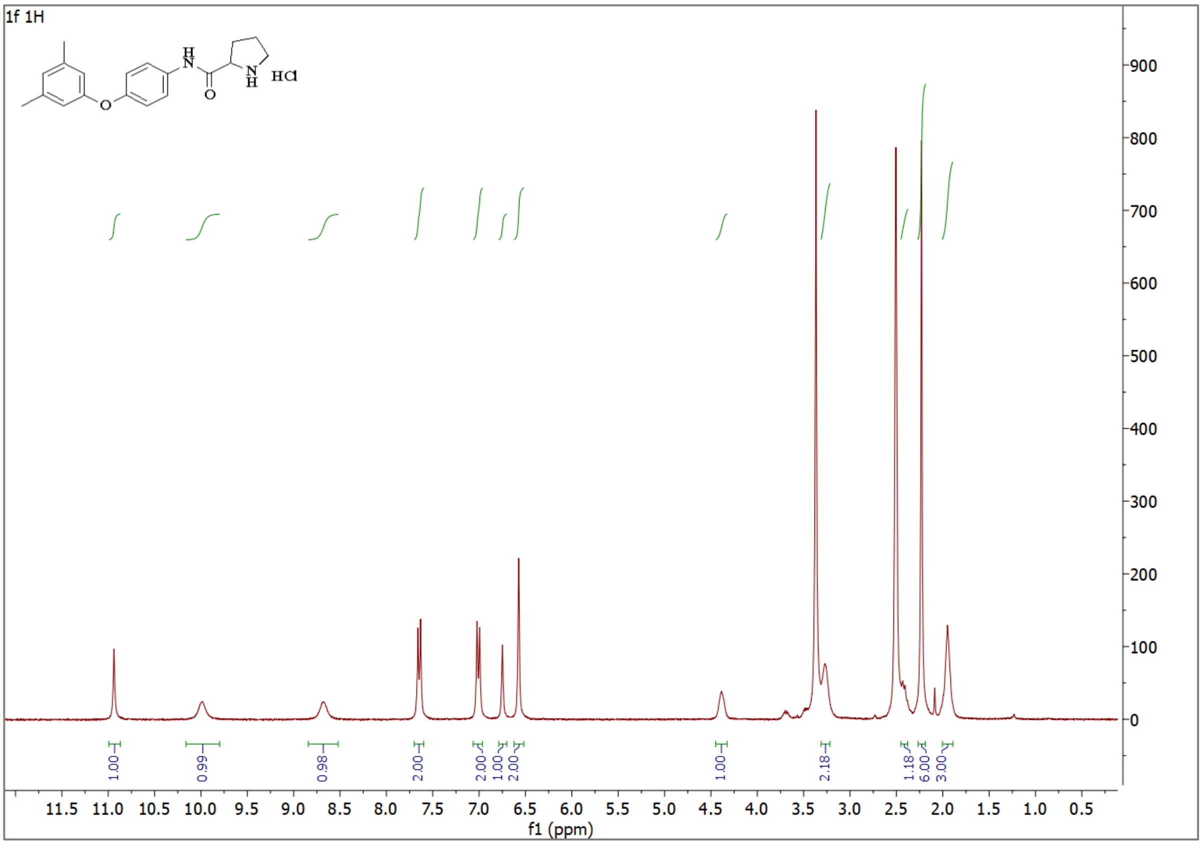

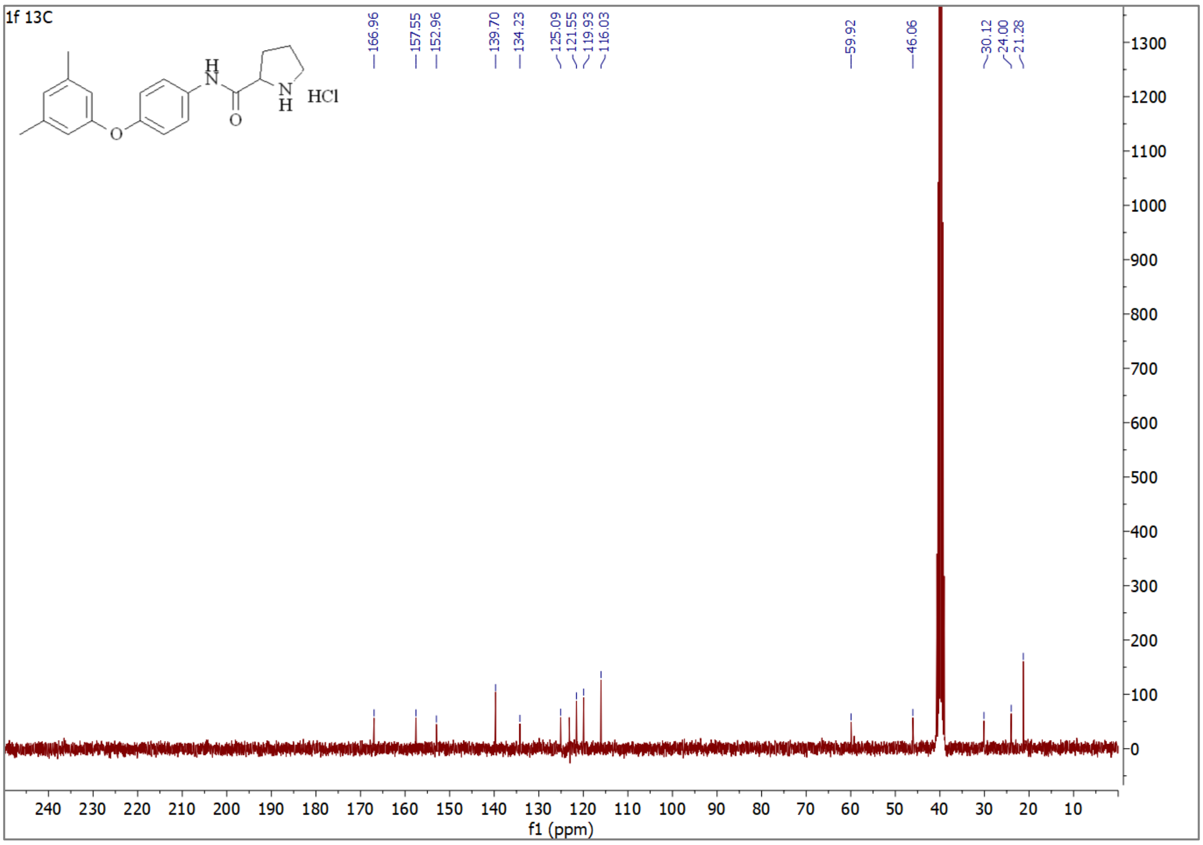

^{1}H and ^{13}C NMR spectra for compound 1g

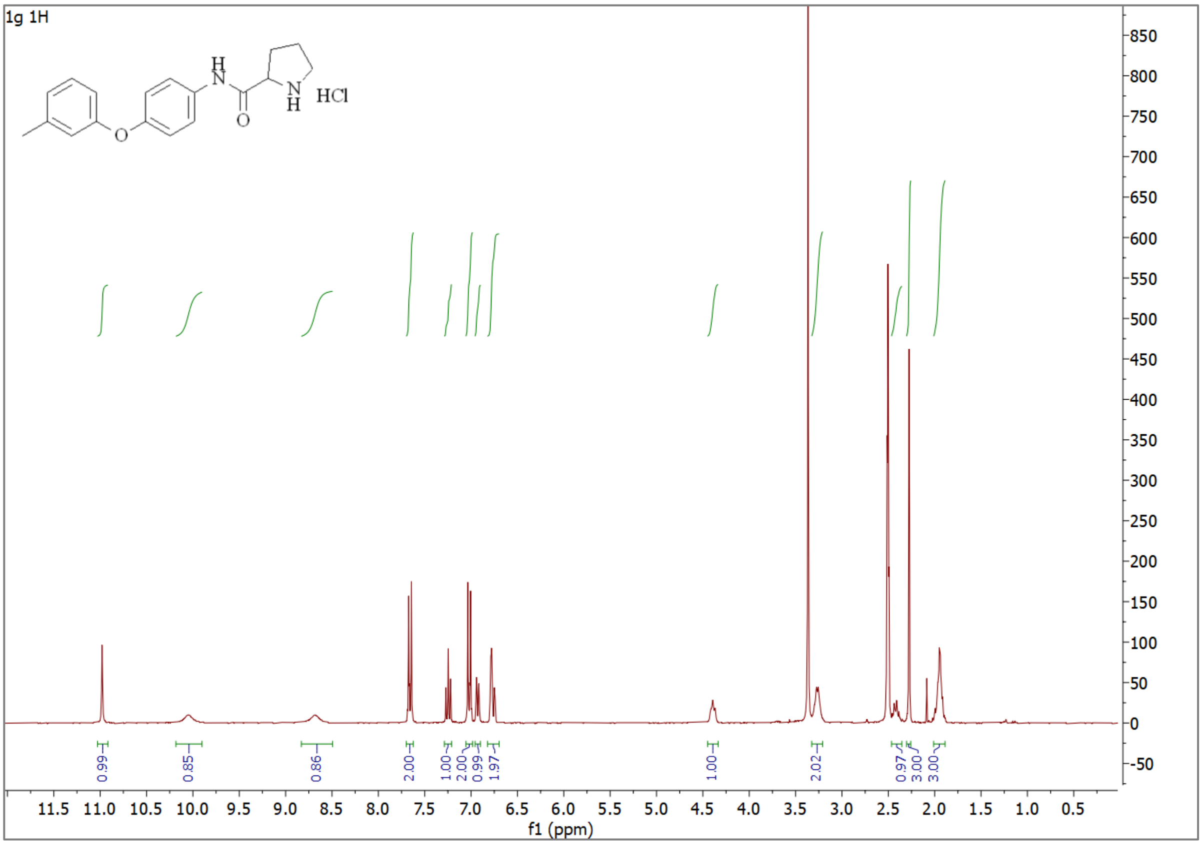

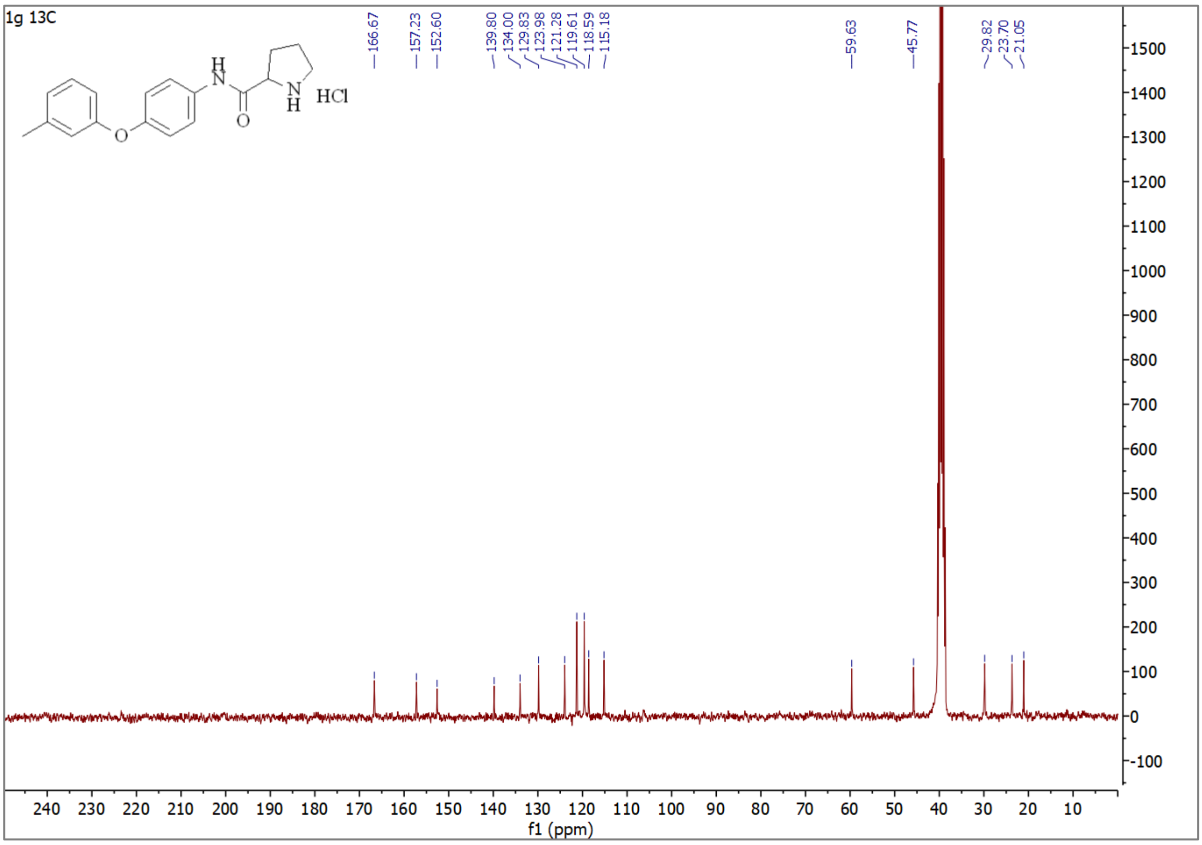

^{1}H and ^{13}C NMR spectra for compound 2a

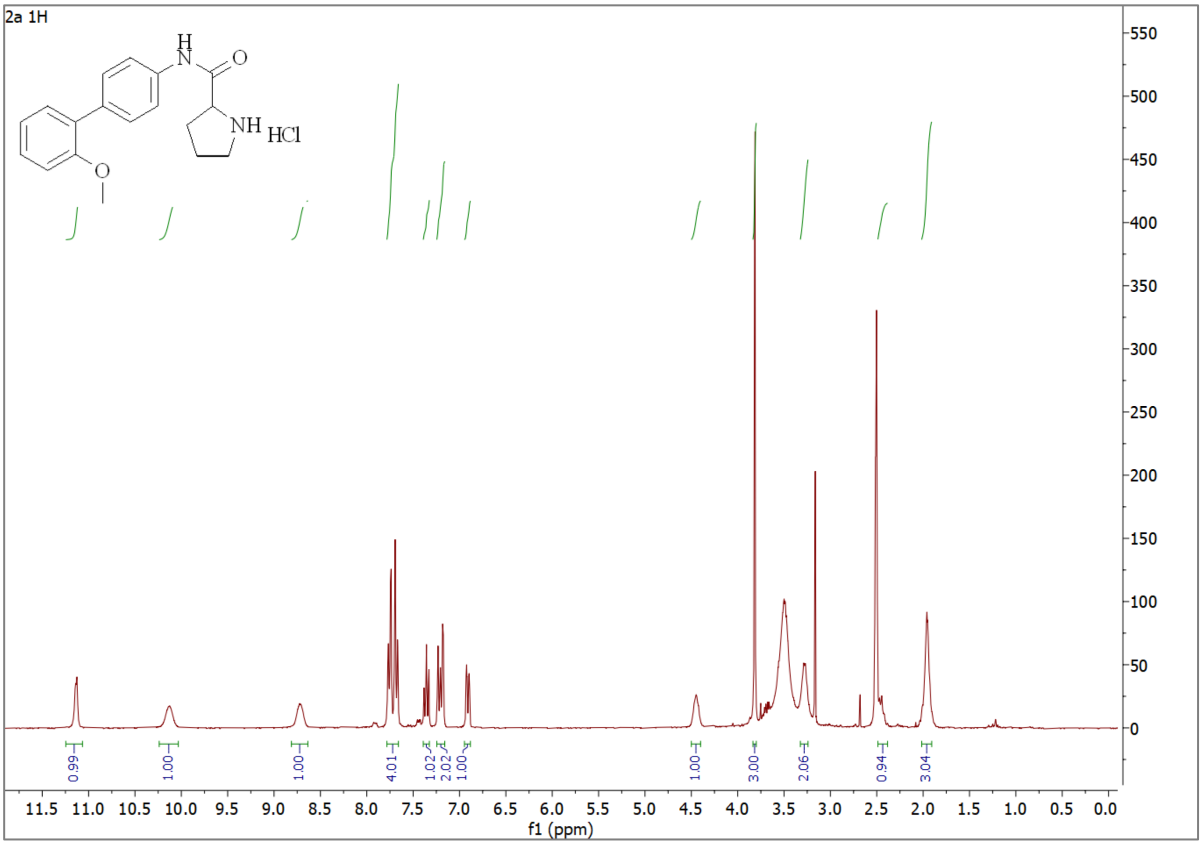

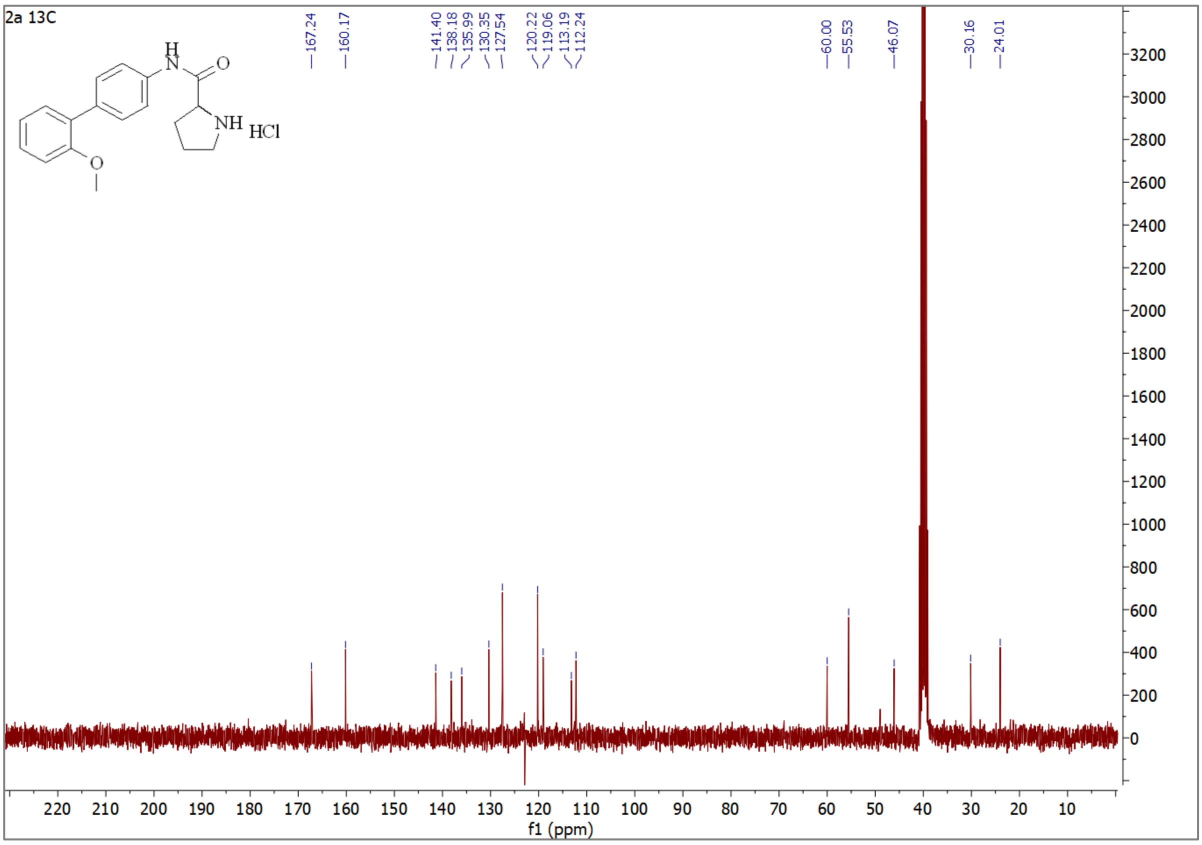

^{1}H and ^{13}C NMR spectra for compound 2b

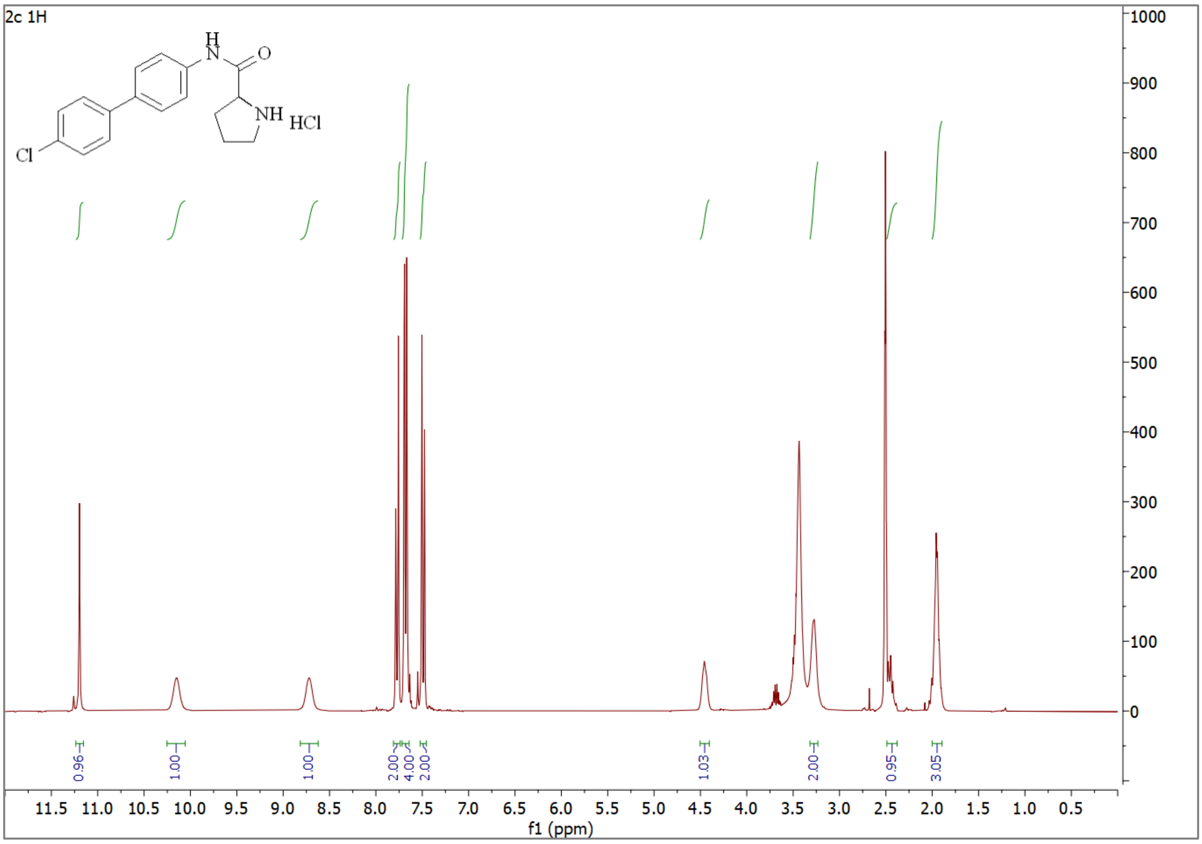

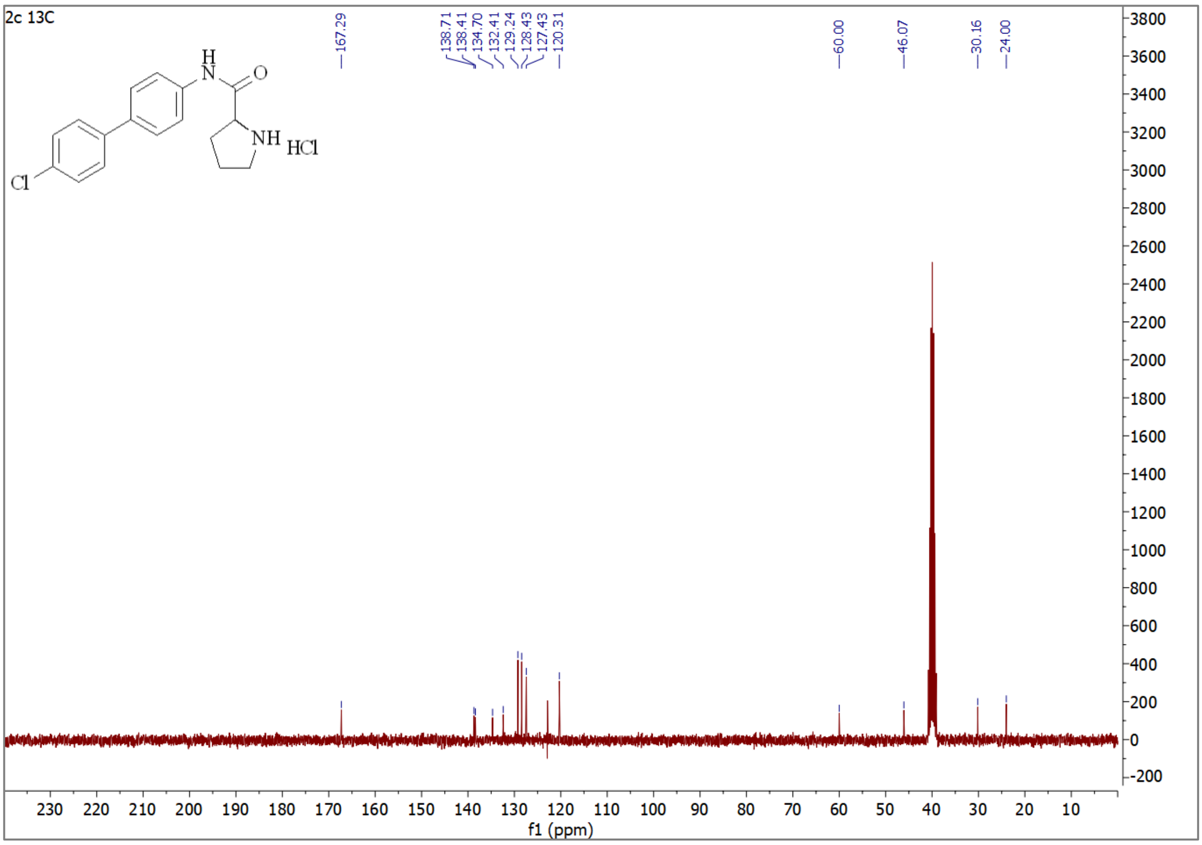

1H and 13C NMR spectra for compound 2c

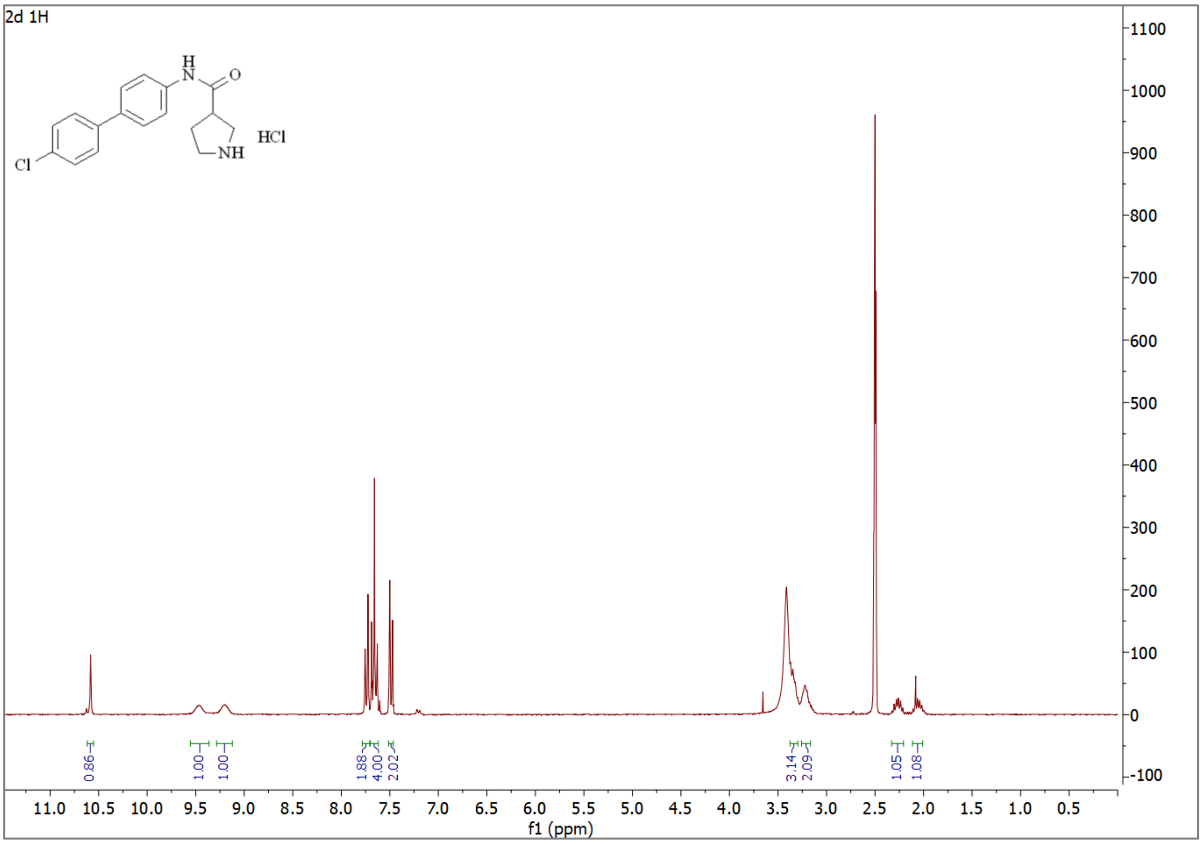

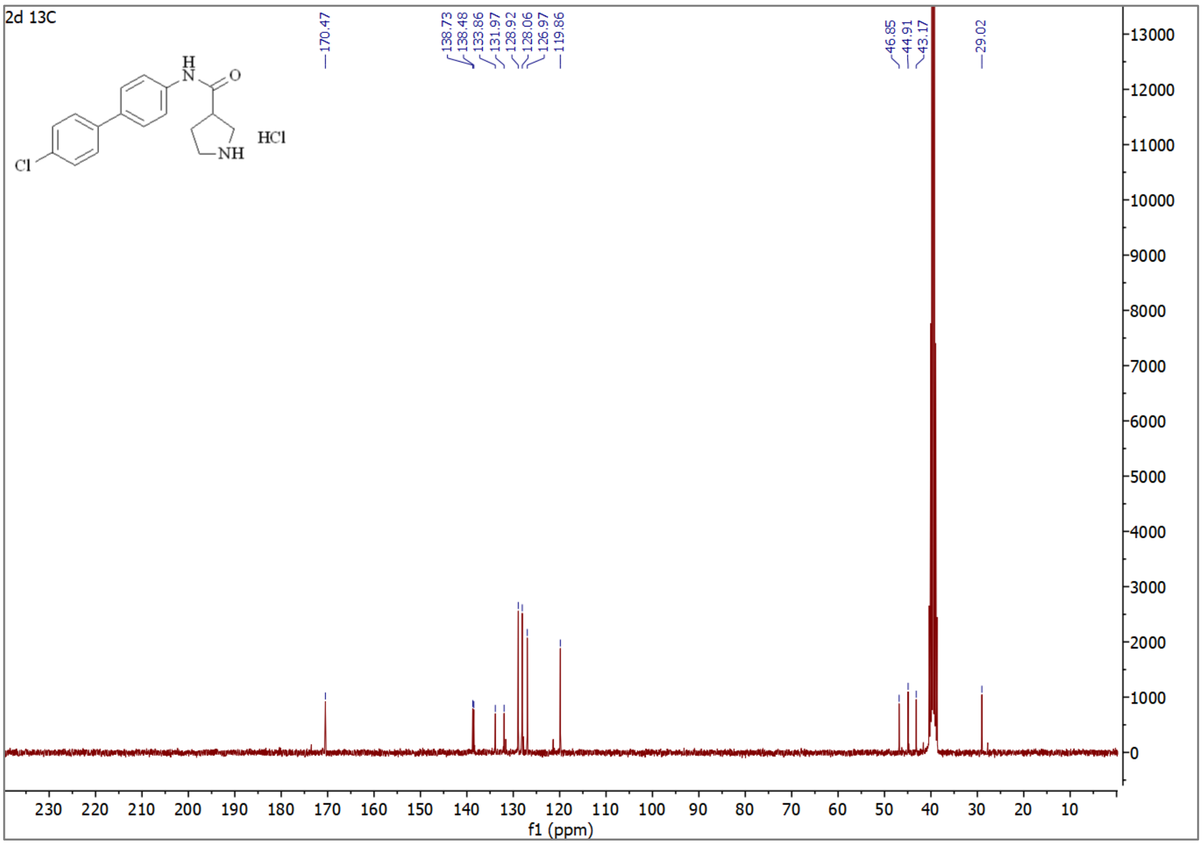

^{1}H and ^{13}C NMR spectra for compound 2d

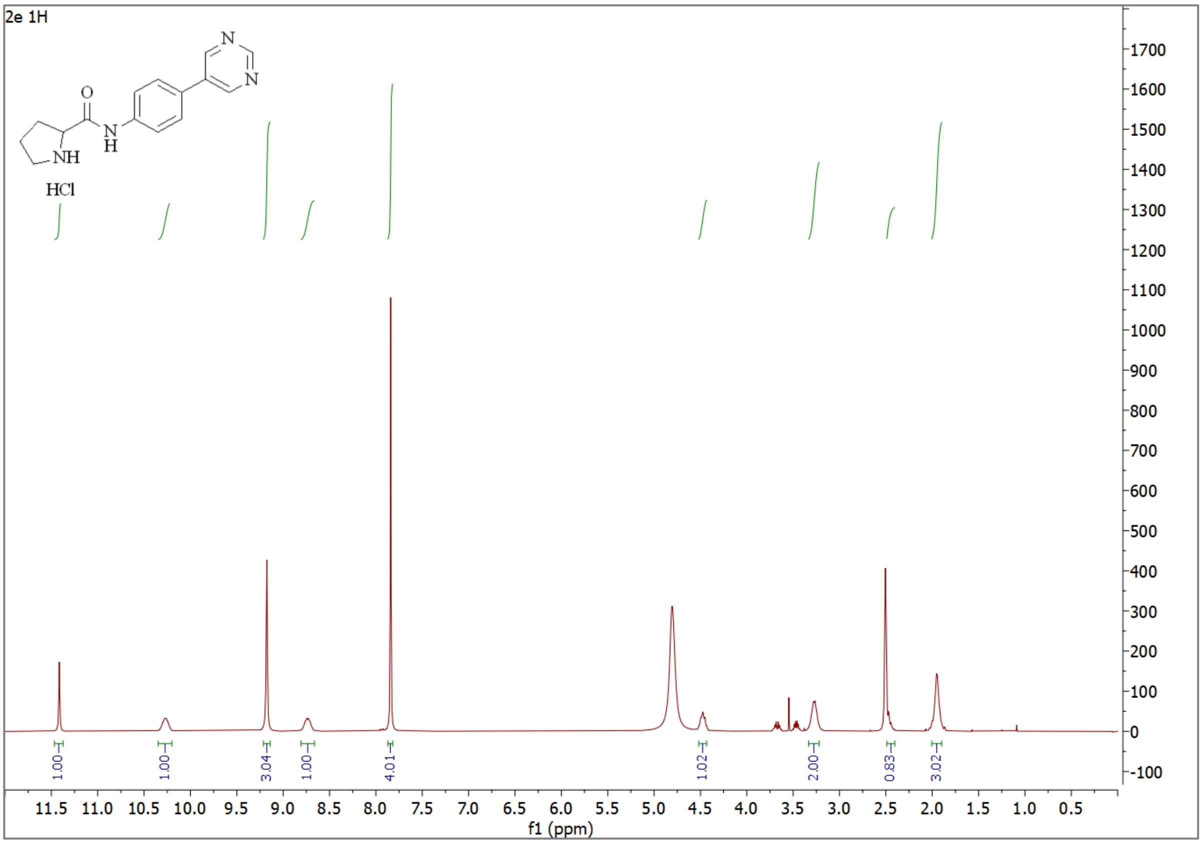

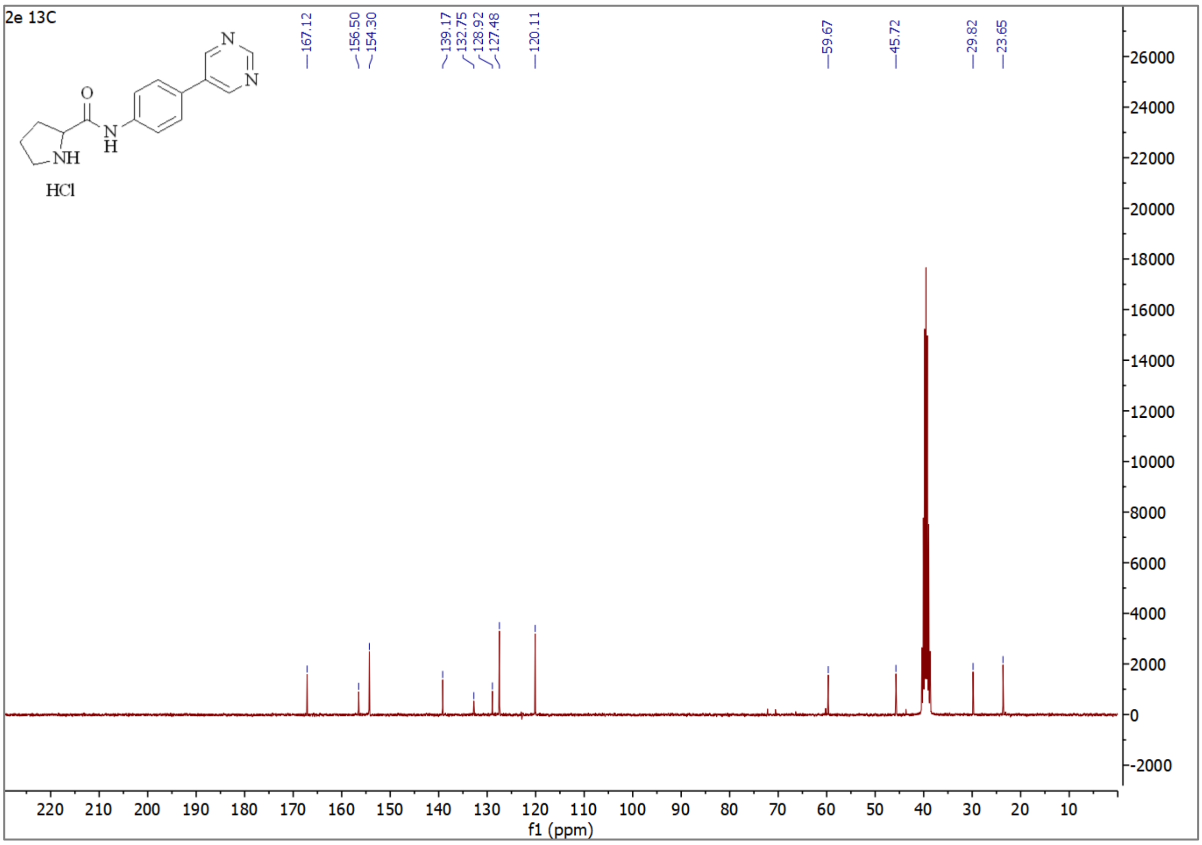

^{1}H and ^{13}C NMR spectra for compound 2e

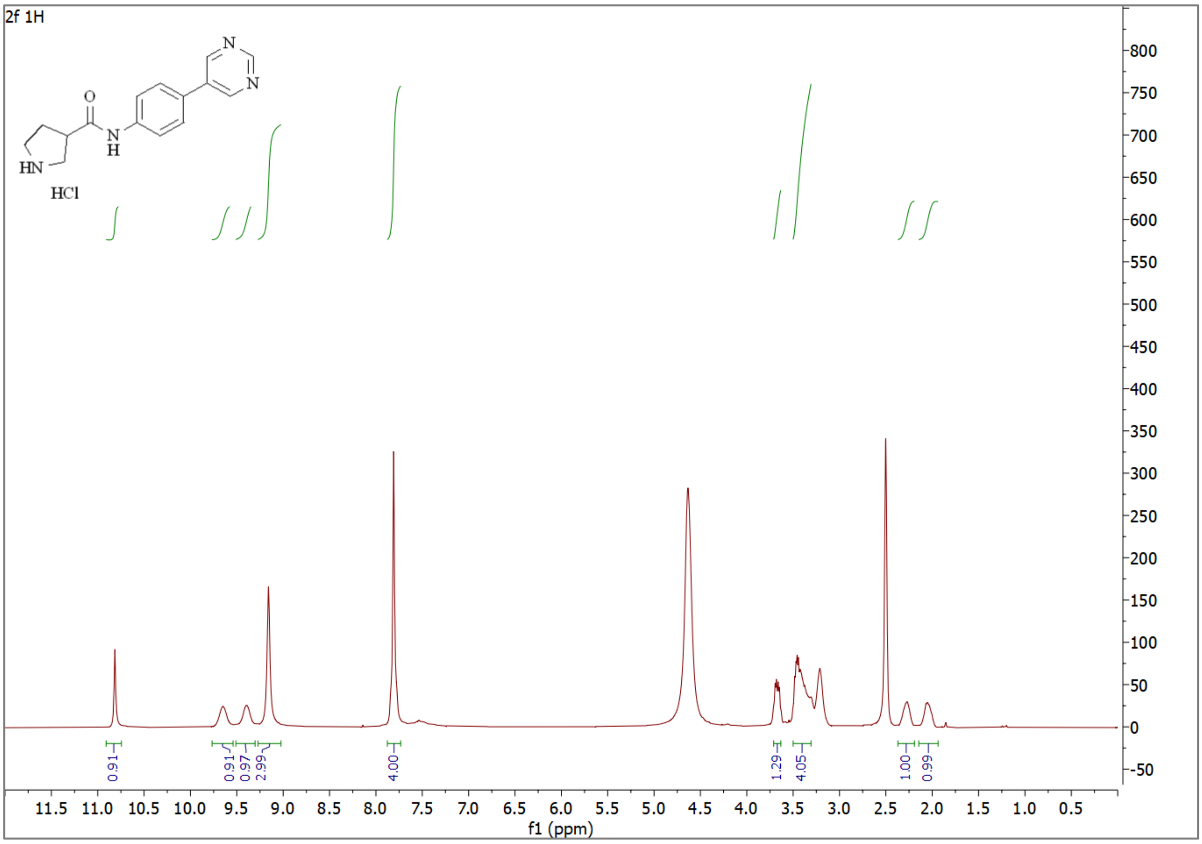

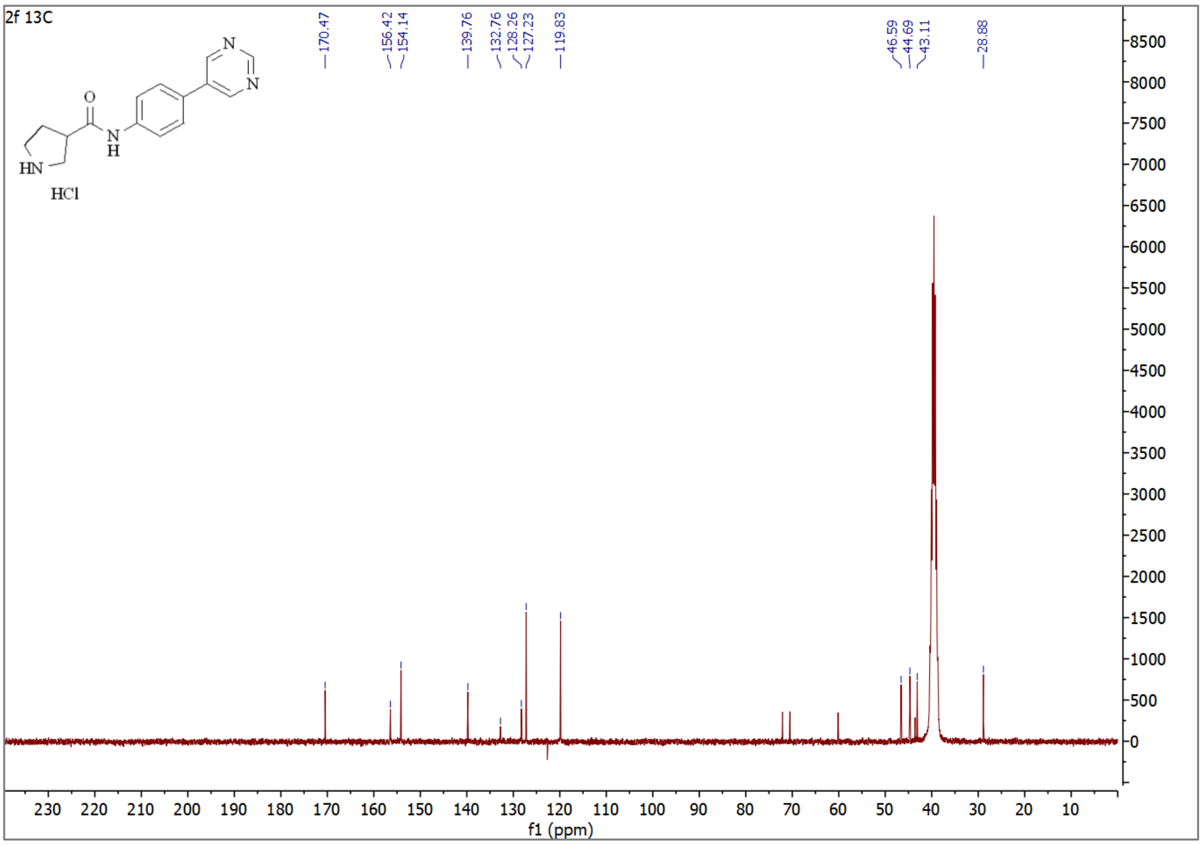

^{1}H and ^{13}C NMR spectra for compound 2f

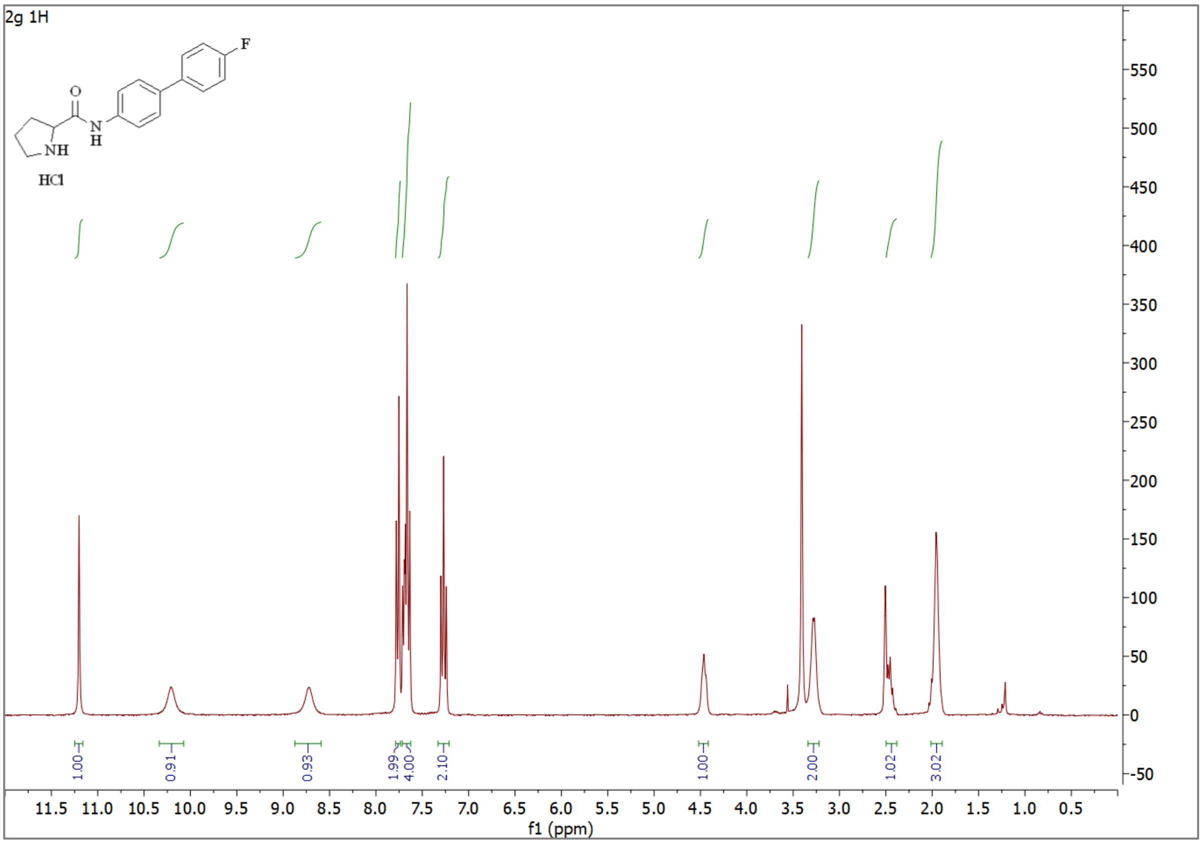

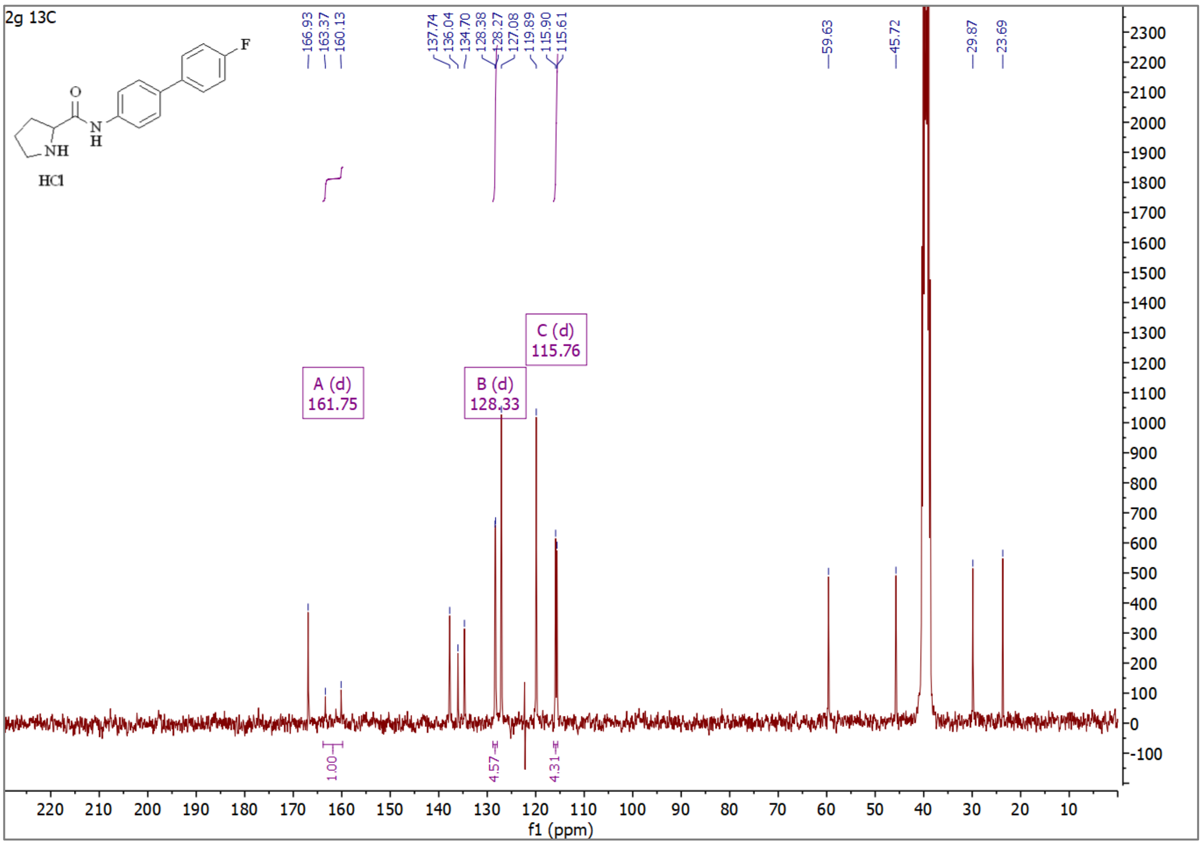

^1H and ^{13}C NMR spectra for compound 2g

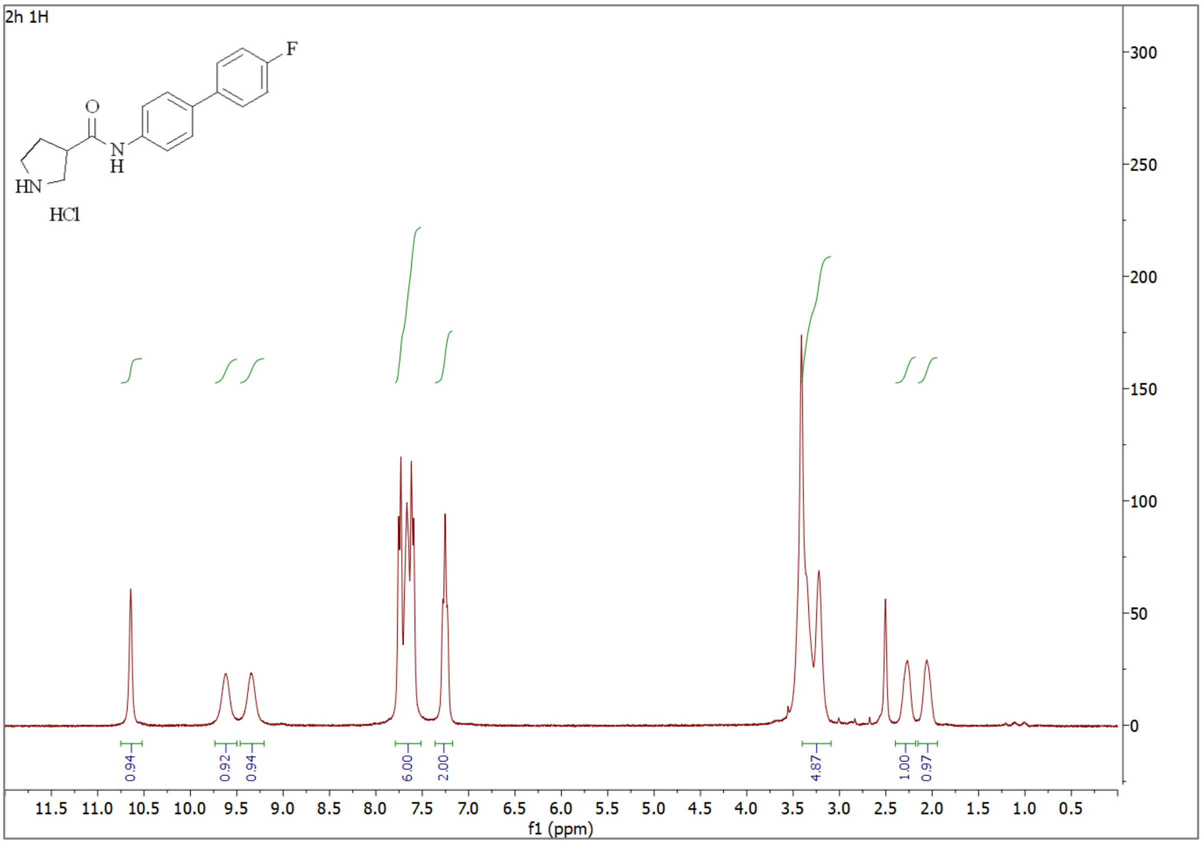

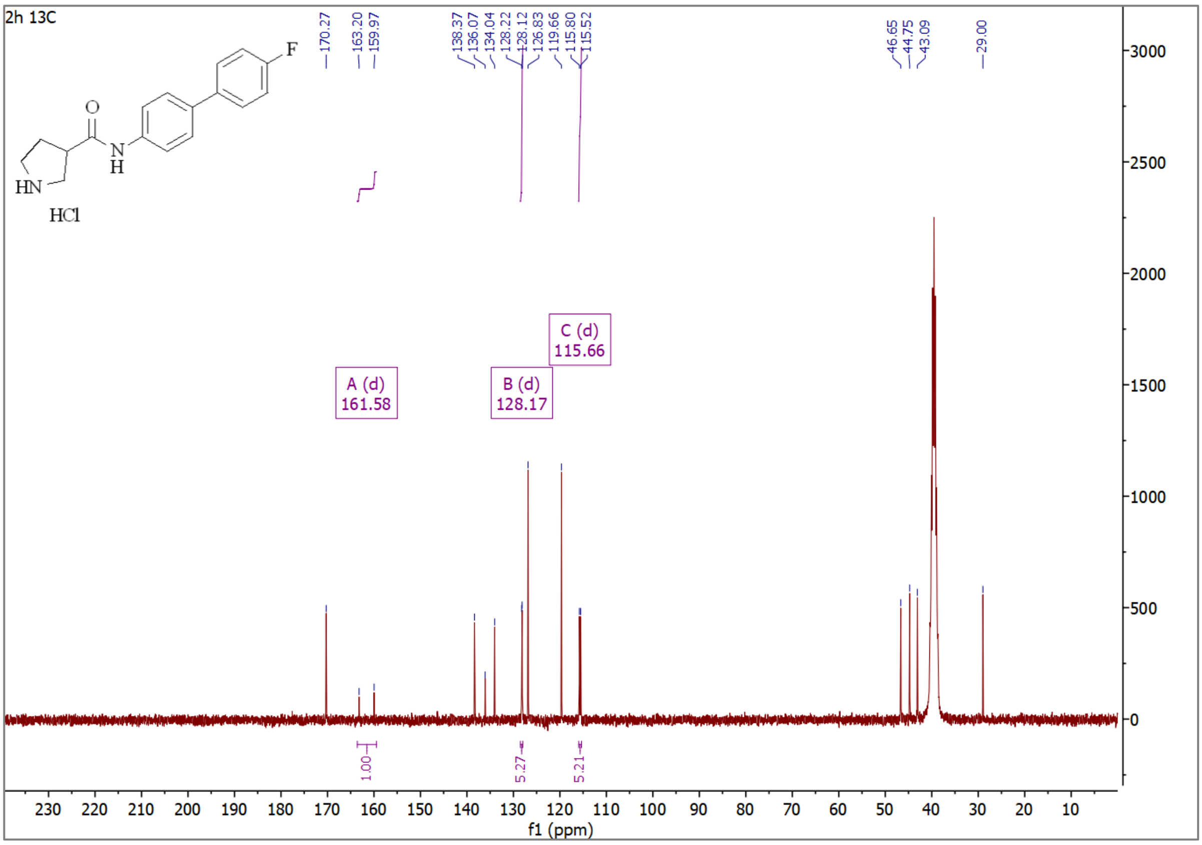

^{1}H and ^{13}C NMR spectra for compound 2h

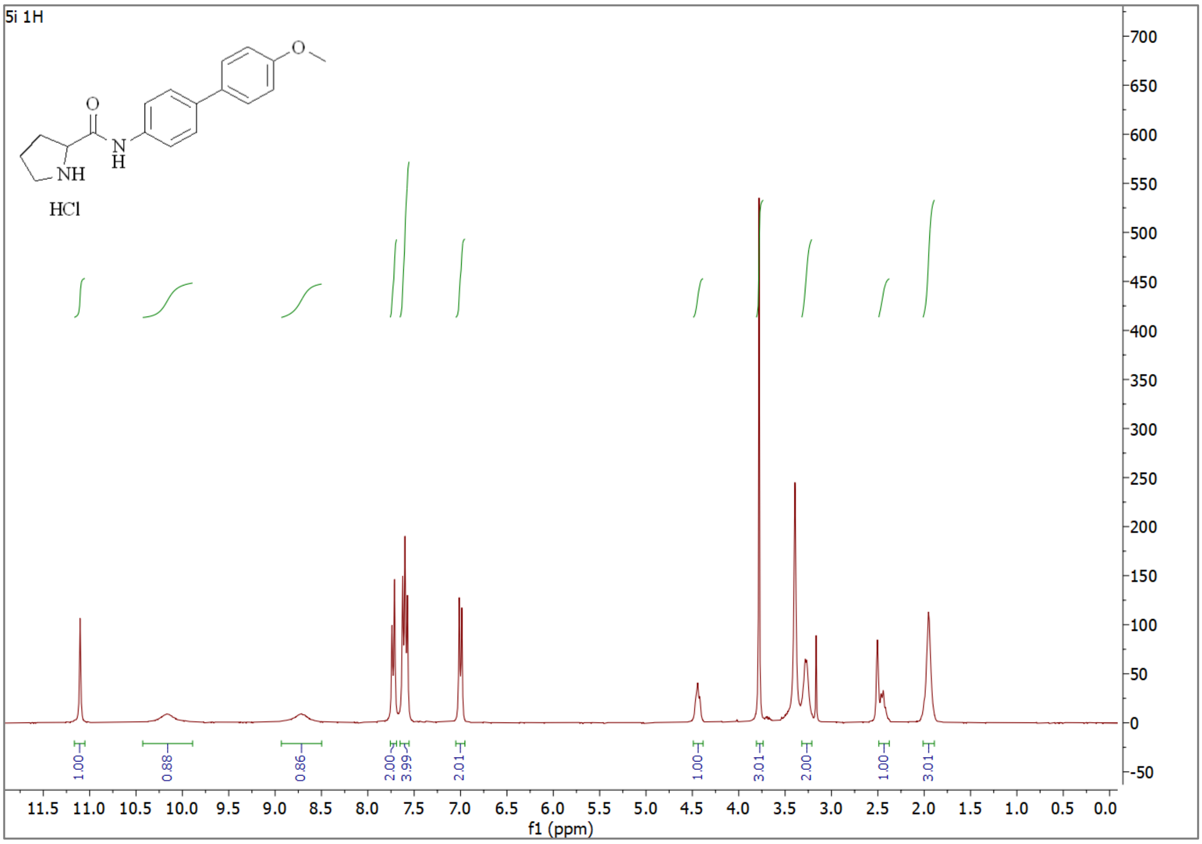

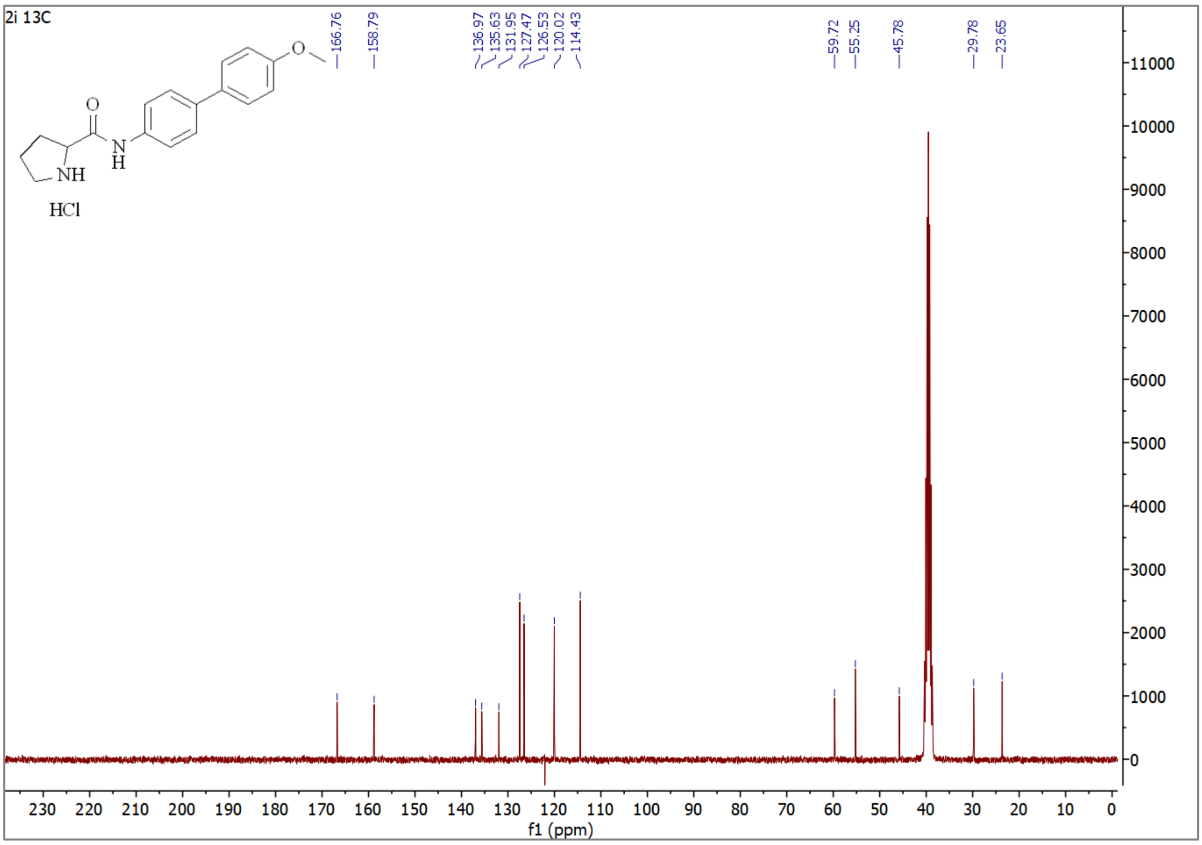

^{1}H and ^{13}C NMR spectra for compound 2i

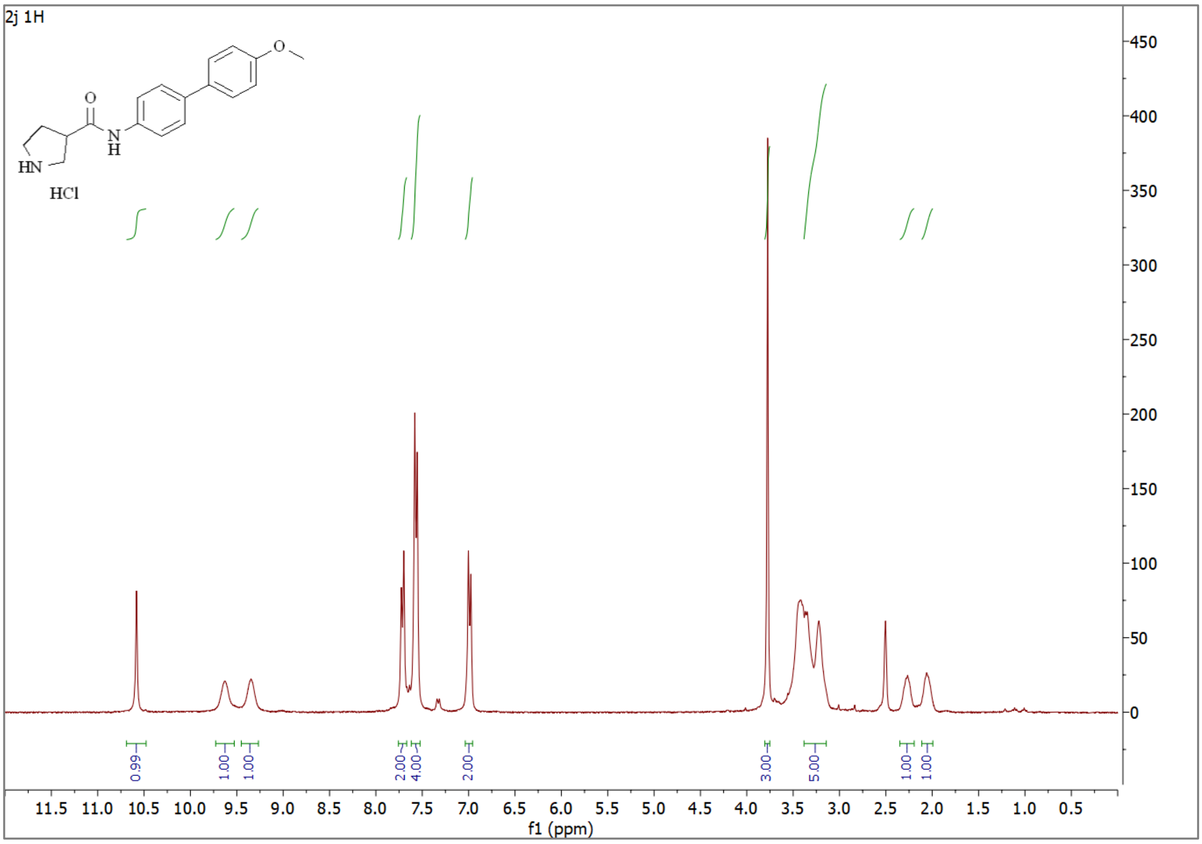

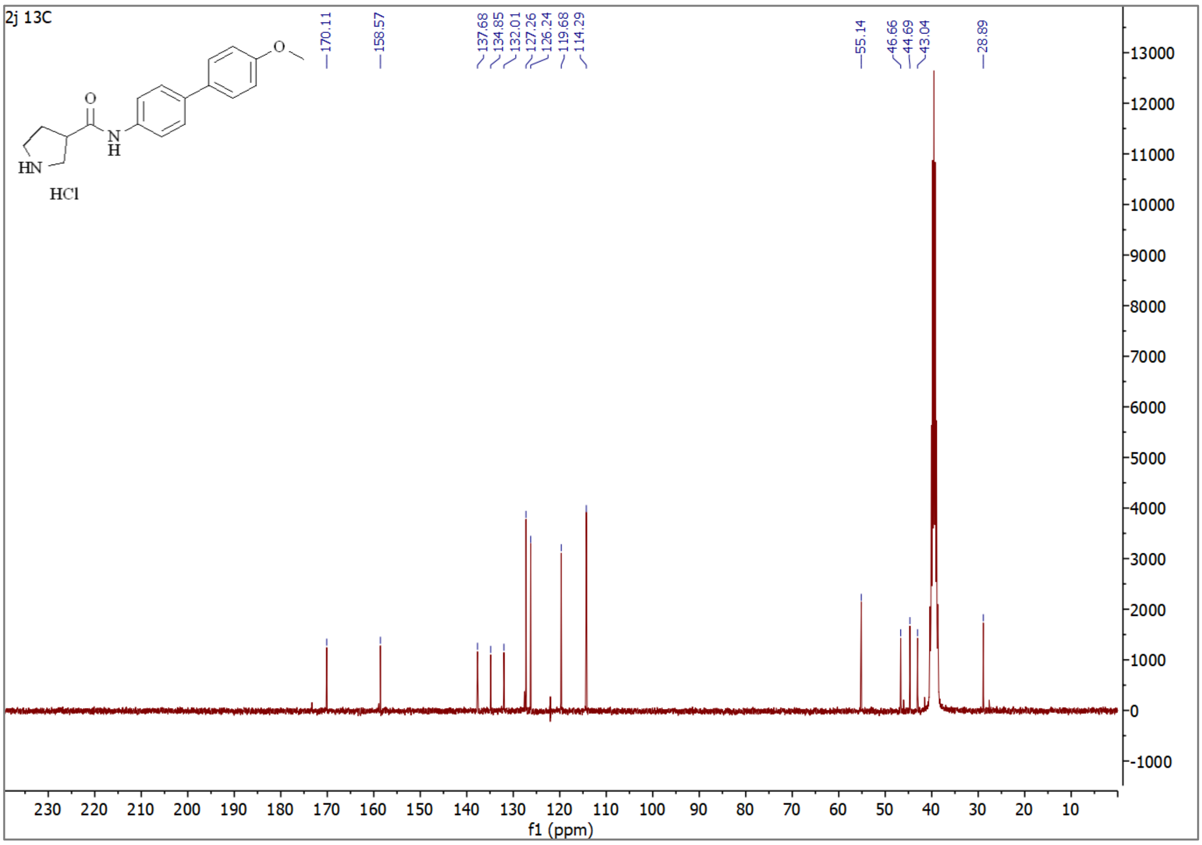

^{1}H and ^{13}C NMR spectra for compound 5a

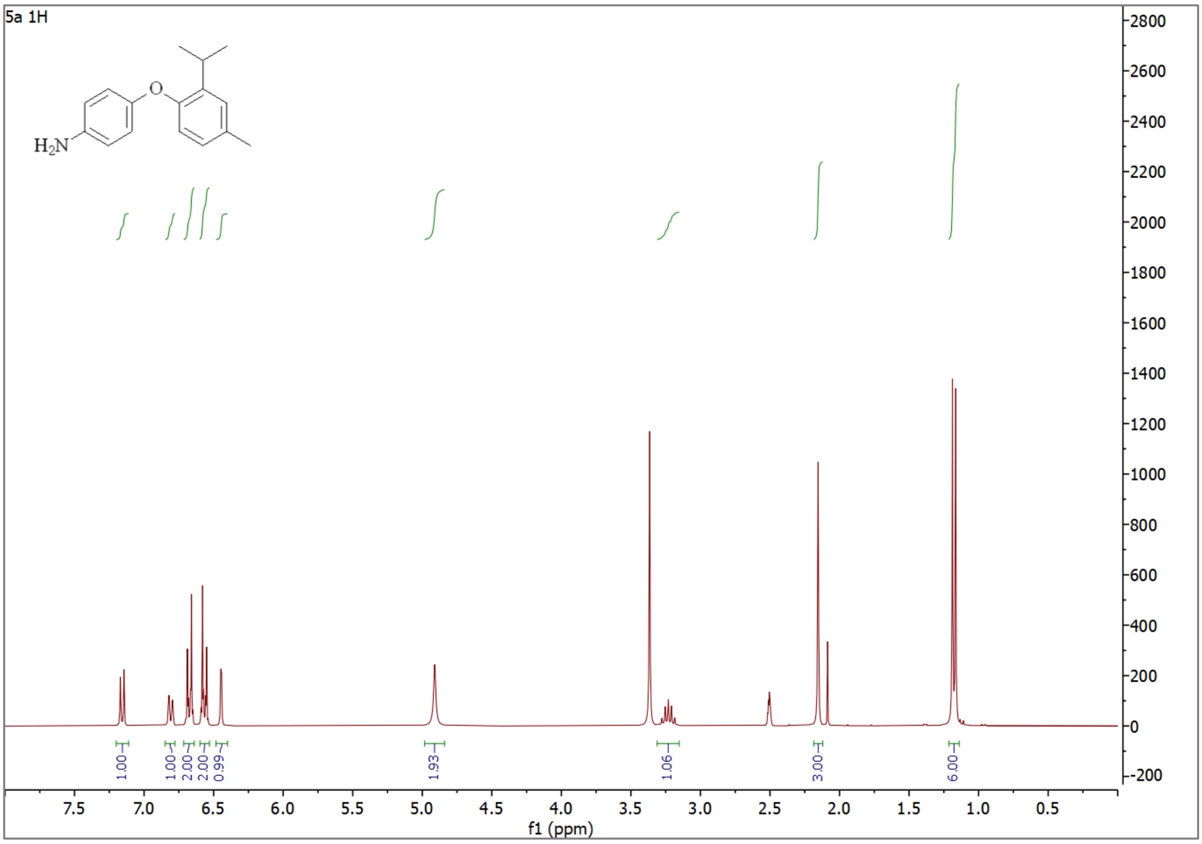

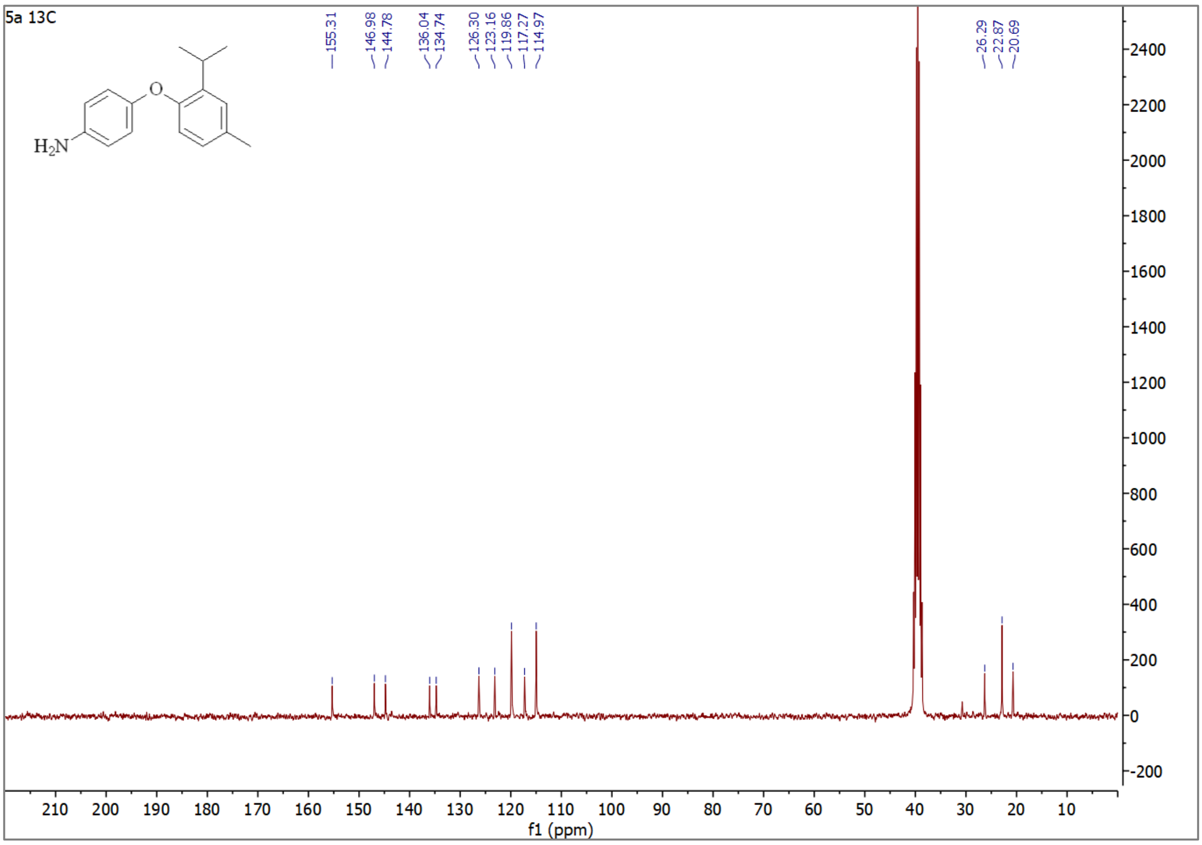

^{1}H and ^{13}C NMR spectra for compound 5b

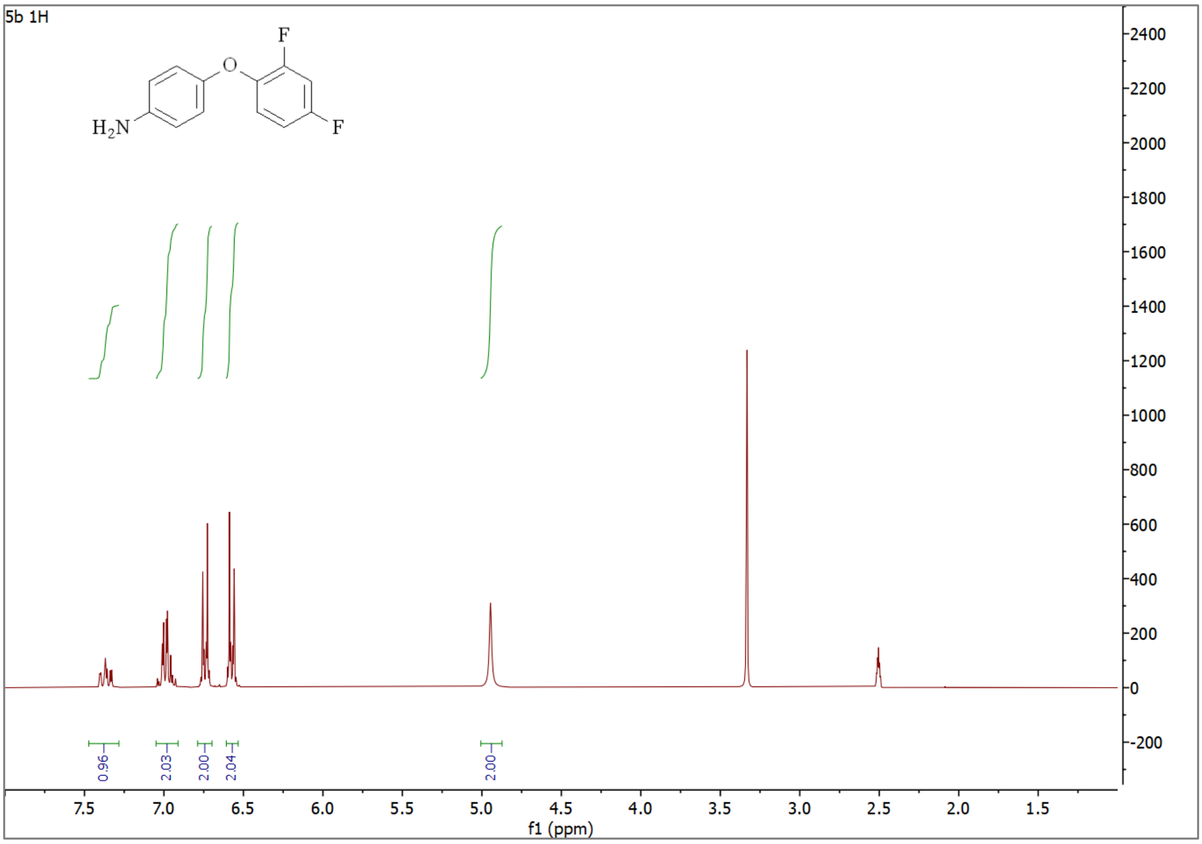

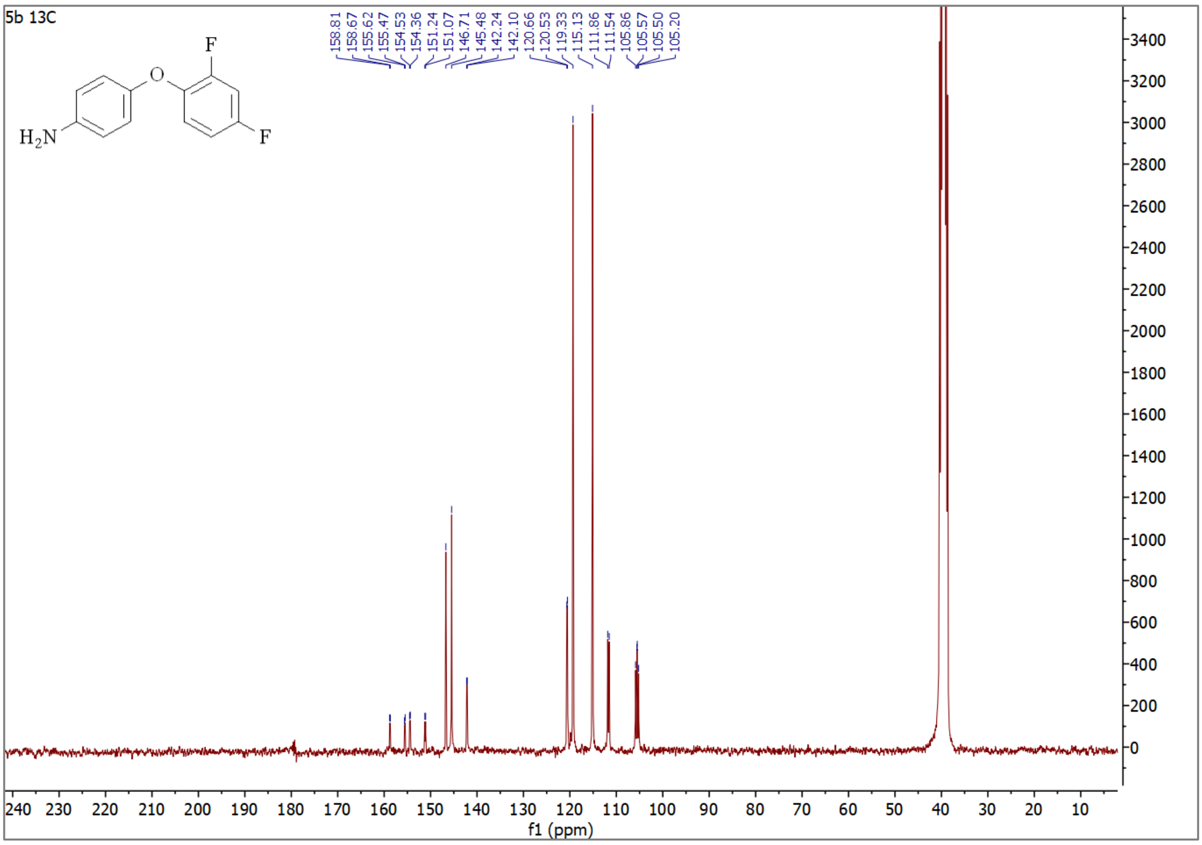

^{1}H and ^{13}C NMR spectra for compound 5c

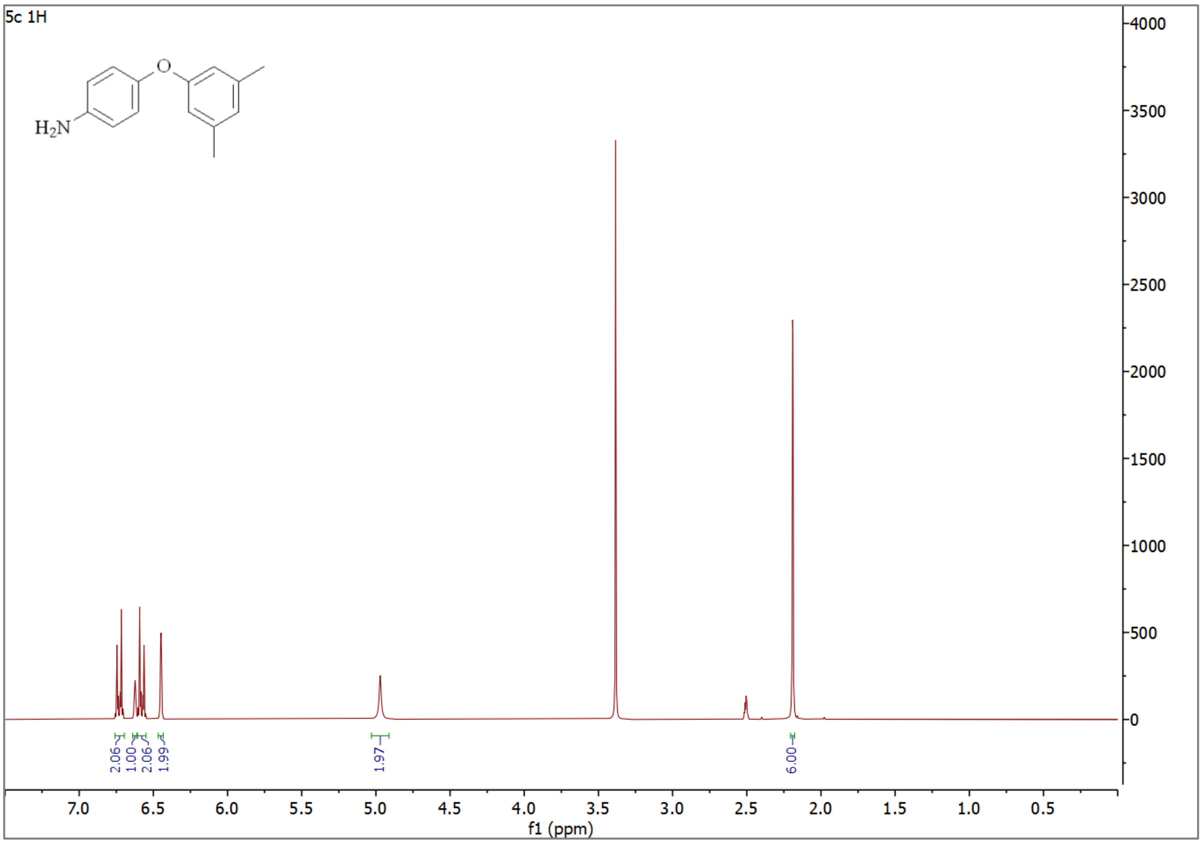

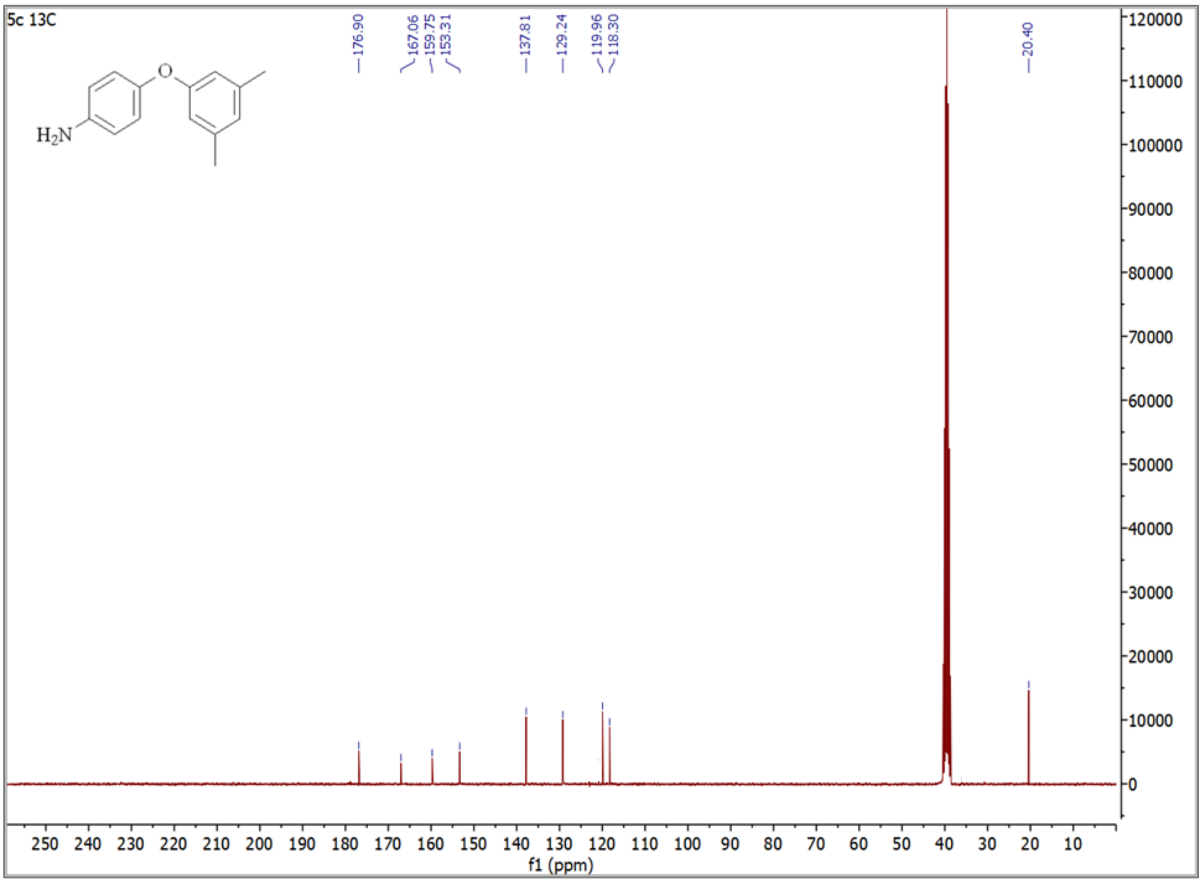

^{1}H and ^{13}C NMR spectra for compound 5d

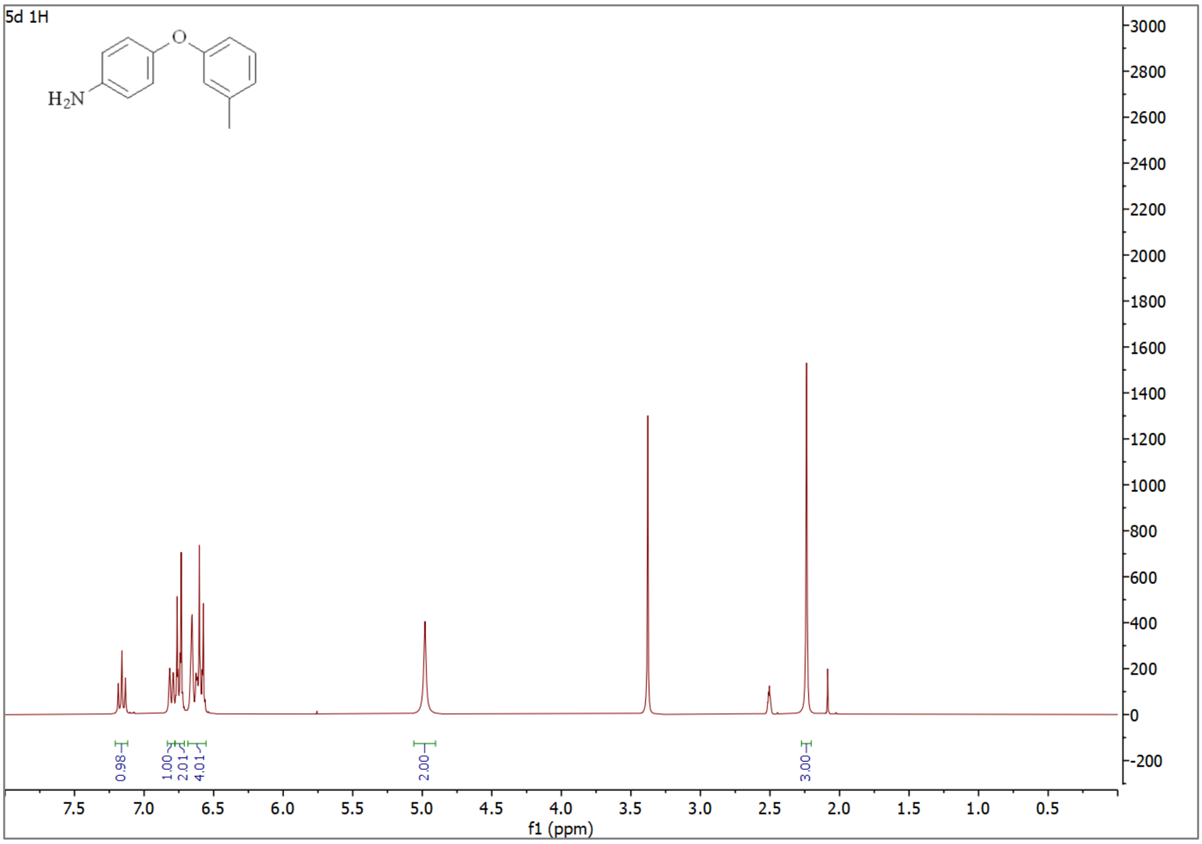

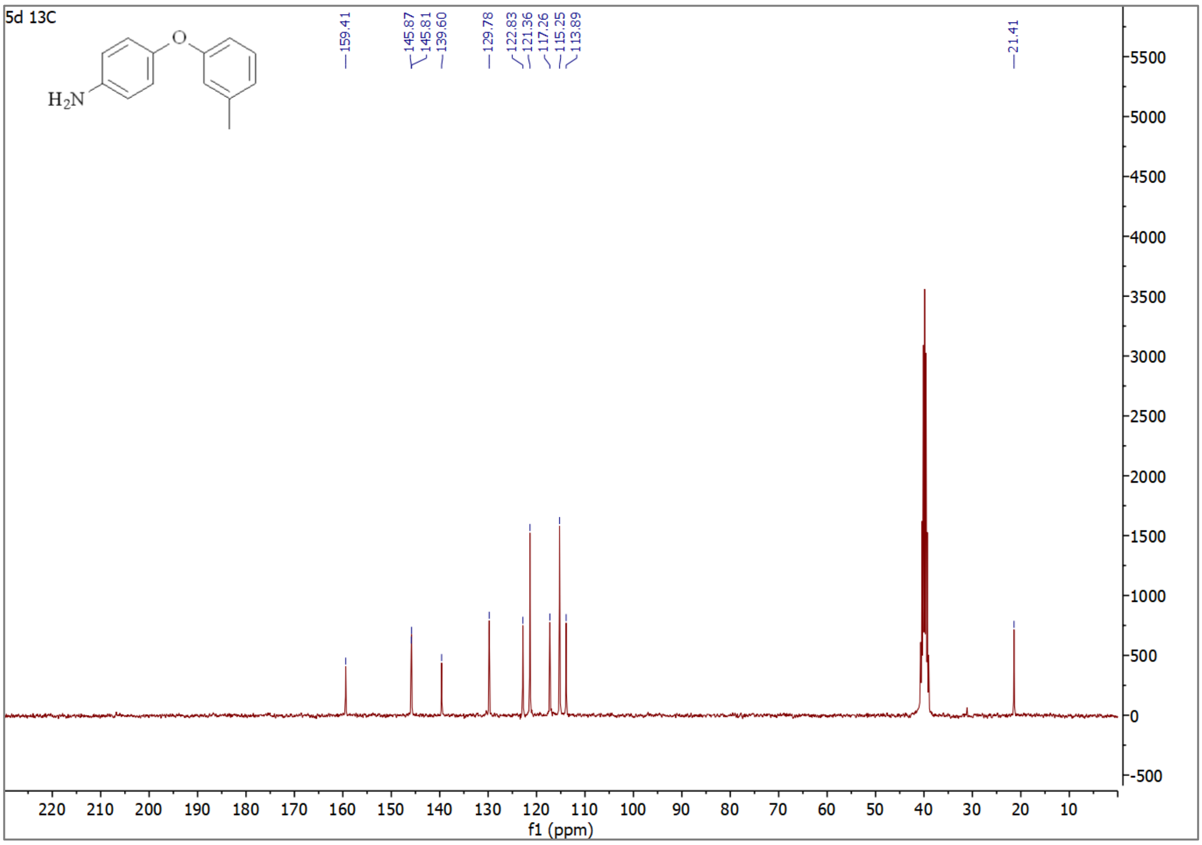

Supplement: Supplementary file 1 [file molecules-31-00240-s001.zip › molecules-4032495-supplementary.pdf]
